# A register and questionnaire study of long-term general health symptoms following SARS-CoV-2 vaccination in Denmark

**DOI:** 10.1038/s41541-024-00844-w

**Published:** 2024-03-04

**Authors:** Elisabeth O’Regan, Ingrid Bech Svalgaard, Anna Irene Vedel Sørensen, Lampros Spiliopoulos, Peter Bager, Nete Munk Nielsen, Jørgen Vinsløv Hansen, Anders Koch, Inger Kristine Meder, Poul Videbech, Steen Ethelberg, Anders Hviid

**Affiliations:** 1 Department of Epidemiology Research, Statens Serum Institut, 2300 Copenhagen, Denmark; 2 Infectious Disease Epidemiology and Prevention, Statens Serum Institut, 2300 Copenhagen, Denmark; 3 Focused Research Unit in Neurology, Department of Neurology, Hospital of Southern Jutland, University of Southern Denmark, 6200 Aabenraa, Denmark; 4 Department of Public Health, Global Health Section, University of Copenhagen, Copenhagen, Denmark; 5 Department of Infectious Diseases, Rigshospitalet University Hospital, 2100 Copenhagen, Denmark; 6 Center for Neuropsychiatric Depression Research, Mental Health Center Glostrup, 2600 Glostrup, Denmark and University of Copenhagen, Copenhagen, Denmark; 7 Pharmacovigilance Research Centre, Department of Drug Design and Pharmacology, University of Copenhagen, 2100 Copenhagen, Denmark

**Keywords:** Epidemiology, Signs and symptoms

## Abstract

Many individuals who refuse COVID-19 vaccination have concerns about long-term side effects. Here, we report findings on self-reported symptoms from a Danish survey- and register study. The study included 34,868 vaccinated primary course recipients, 95.8% of whom received mRNA vaccines, and 1,568 unvaccinated individuals. Participants had no known history of SARS-CoV-2 infection. Using g-computation on logistic regression, risk differences (RDs) for symptoms between vaccinated and unvaccinated persons were estimated with adjustments for possible confounders. Within six weeks after vaccination, higher risks were observed for physical exhaustion (RD 4.9%, 95% CI 1.1% to 8.4%), fever or chills (RD 4.4%, 95% CI 2.1% to 6.7%), and muscle/joint pain (RD 7.0%, 95% CI 3.1% to 10.7%), compared to unvaccinated individuals. Beyond twenty-six weeks, risks were higher among the vaccinated for sleeping problems (RD 3.0, 95% 0.2 to 5.8), fever or chills (RD 2.0, 95% CI 0.4 to 3.6), reduced/altered taste (RD 1.2, 95% CI 0.2 to 2.3) and shortness of breath (RD 2.6, 95% CI 0.9 to 4.0). However, when examining pre-omicron responses only, the difference for reduced/altered taste was significant. As expected, the risk of experiencing physical exhaustion, fever or chills, and muscle/joint pain was higher among persons who responded within six weeks of completing the primary course. No significant differences were observed for the 7-25-week period after vaccination. Associations for the period beyond 26 weeks must be interpreted with caution and in the context of undetected SARS-CoV-2 infection, wide confidence intervals, and multiple testing. Overall, we observe no concerning signs of long-term self-reported physical, cognitive, or fatigue symptoms after vaccination.

## Introduction

Many individuals who refuse COVID-19 vaccines have concerns about possible side effects^1^. Unprecedented efforts have been made to monitor and evaluate the safety of COVID-19 vaccines, which continue to protect against serious illness and death from COVID-19 infection. Indeed, several largescale clinical and randomized trials have gathered that local and systemic reactions to COVID-19 vaccines are common but usually subside within a few days^2–5^. In addition, post-marketing surveillance has led to the detection of rare but serious adverse events occurring in the weeks following COVID-19 vaccination such as myocarditis/pericarditis^6–8^ and thrombosis with thrombocytopenia syndrome (TTS)^9,10^. Still, more research is needed to provide reassurance that COVID-19 vaccines are safe in the long-term.

Anecdotes about COVID-19 vaccines causing long-term side effects have circulated on social media, fueling concerns about vaccine safety^11^. While much survey-based research has been centered around long covid symptoms, insufficient work has used population-level surveys to address the possibility of long-term symptoms following COVID-19 vaccines. Thus far, most COVID-19 vaccine safety research has focused on registered diagnostic outcomes, but many potential issues are not ascertained well in register data alone. Therefore, to further ensure COVID-19 vaccine safety, it is valuable to examine symptom outcomes of COVID-19 vaccines over time by using large-scale, self-reported survey data.

The aim of this study was to explore the safety of primary course COVID-19 vaccines by combining unique Danish register and survey data. By including only individuals with no known history of SARS-CoV-2, we sought to estimate the absolute effect of primary course COVID-19 vaccination on self-reported general health symptoms. These symptoms include self-reported cognitive, fatigue, and physical symptoms.

## Results

### Overview of study population

After exclusion criteria were applied, our sample consisted of 36,436 participants. Vaccinated participants comprised 95.6% (*n* = 34,868) of responses, whereas unvaccinated comprised 4.3% (*n* = 1568). The proportions of vaccinated and unvaccinated were similar across all tracks (Supplementary Table 5). There was a greater proportion of female participants in both unvaccinated (71.0%) and vaccinated (58.6%) groups, and roughly half of participants had a higher education of ≥2 years among both unvaccinated and vaccinated groups (Table 1). Among the vaccinated, 82.4% (*n* = 28,719) had received two doses of BNT162b2, 13.4% (*n* = 4,660) two doses of MRNA-1273, 4.0% (*n* = 1394) ChAdOx1 for dose 1 and an mRNA vaccine for dose 2, 0.2% (*n* = 78) one dose of Ad26.COV2.S, 0.04% (*n* = 13) two doses of ChAdOx1, and 0.01% (*n* = 4) mixed mRNA vaccines (Supplementary Fig. 3, Panel B). Most respondents (87.4%) completed their follow-up questionnaire 7–25 weeks after completion of the primary course with a median time since vaccination of 19.1 weeks (Supplementary Fig. 3, Panel C, Supplementary Fig. 2). Among the respondents who were vaccinated less than six weeks and more than 26 weeks prior to responding to the follow-up questionnaire, the median time since vaccination was 4.6 weeks and 28.6 weeks, respectively. Responses among unvaccinated participants remained fairly stable over time, whereas those who reported symptoms >26 weeks after vaccination mostly responded during the months of November and December 2021 (Supplementary Fig. 4). Compared to non-respondents, survey participants were more often female, older, and had more comorbidities (Supplementary Table 6)^12^.

**Table 1:** Characteristics of 36,436 EFTER COVID survey participants who tested negative for SARS-Cov-2

	Unvaccinated	Vaccinated	*p* value
	(*N* = 1568)	(*N* = 34868)	
**Age (years)**			
15–19	22 (1.4%)	917 (2.6%)	≤0.001
20–29	168 (10.7%)	2661 (7.6%)	
30–39	249 (15.9%)	4221 (12.1%)	
40–49	324 (20.7%)	6514 (18.7%)	
50–59	442 (28.2%)	10123 (29.0%)	
60–69	268 (17.1%)	7849 (22.5%)	
70+	95 (6.1%)	2583 (7.4%)	
**Sex**			
Female	1114 (71.0%)	20449 (58.6%)	≤0.001
Male	454 (29.0%)	14419 (41.4%)	
**Origin**			
Danish	1326 (84.6%)	32538 (93.3%)	≤0.001
Born abroad	219 (14.0%)	2144 (6.1%)	
Second generation in Denmark	23 (1.5%)	186 (0.5%)	
**Education**			
Higher education (>5 years, MSc, PhD)	220 (14.0%)	7036 (20.2%)	≤0.001
Higher education (2–4 years, BsC)	544 (34.7%)	11223 (32.2%)	
Higher education (1–2 years, vocational academy)	207 (13.2%)	4190 (12.0%)	
General secondary or vocational secondary education	147 (9.4%)	2845 (8.2%)	
Vocational training	286 (18.2%)	6505 (18.7%)	
Primary or elementary school (9^th^-10^th^ grade)	124 (7.9%)	2547 (7.3%)	
Don’t know	40 (2.6%)	522 (1.5%)	
**Employment**			
Employed full-time	729 (46.5%)	20578 (59.0%)	≤0.001
Employed part-time	201 (12.8%)	3003 (8.6%)	
Self-employed	168 (10.7%)	1719 (4.9%)	
Student	105 (6.7%)	2005 (5.8%)	
Benefits recipient	10 (0.6%)	66 (0.2%)	
Long-term sick leave	30 (1.9%)	290 (0.8%)	
Pensioner	204 (13.0%)	5179 (14.9%)	
Stay-at-home parent or on parental leave	26 (1.7%)	340 (1.0%)	
Unemployed	37 (2.4%)	522 (1.5%)	
Other	58 (3.7%)	1166 (3.3%)	
**Obesity**			
No	1352 (86.2%)	28530 (81.8%)	≤0.001
Yes	216 (13.8%)	6338 (18.2%)	
**Alcohol consumption**			
Moderate (0–10 units per week)	815 (52.0%)	24166 (69.3%)	≤0.001
Heavy (>10 units per week)	87 (5.5%)	3295 (9.5%)	
Never	666 (42.5%)	7407 (21.2%)	
**Smoking**			
Daily (≥10 cigarettes per day)	131 (8.4%)	1734 (5.0%)	≤0.001
Daily (<10 cigarettes per day)	97 (6.2%)	1522 (4.4%)	
Occasionally	93 (5.9%)	2034 (5.8%)	
Electronic cigarettes	28 (1.8%)	404 (1.2%)	
Yes, within the past five years	93 (5.9%)	1548 (4.4%)	
Not within the past five years	364 (23.2%)	9377 (26.9%)	
Never	762 (48.6%)	18249 (52.3%)	
**Charlson comorbidity score**			
0	1422 (90.7%)	31016 (89.0%)	0.171
1	75 (4.8%)	2044 (5.9%)	
2	58 (3.7%)	1431 (4.1%)	
3 or more	13 (0.8%)	377 (1.1%)	

Questionnaires were filled out between August 1, 2021 and May 5, 2022.

### Symptom prevalence and risk differences

When examining the prevalence of outcomes for different age groups and sex regardless of vaccination status, we observed that there was a high prevalence of self-reported mental exhaustion among young people (aged 15-29 years) (Supplementary Fig. 5, Panel A). Overall, regardless of vaccination status and age group, females had a higher prevalence of self-reported symptoms compared to males (Supplementary Fig. 5).

**Fig. 1:**
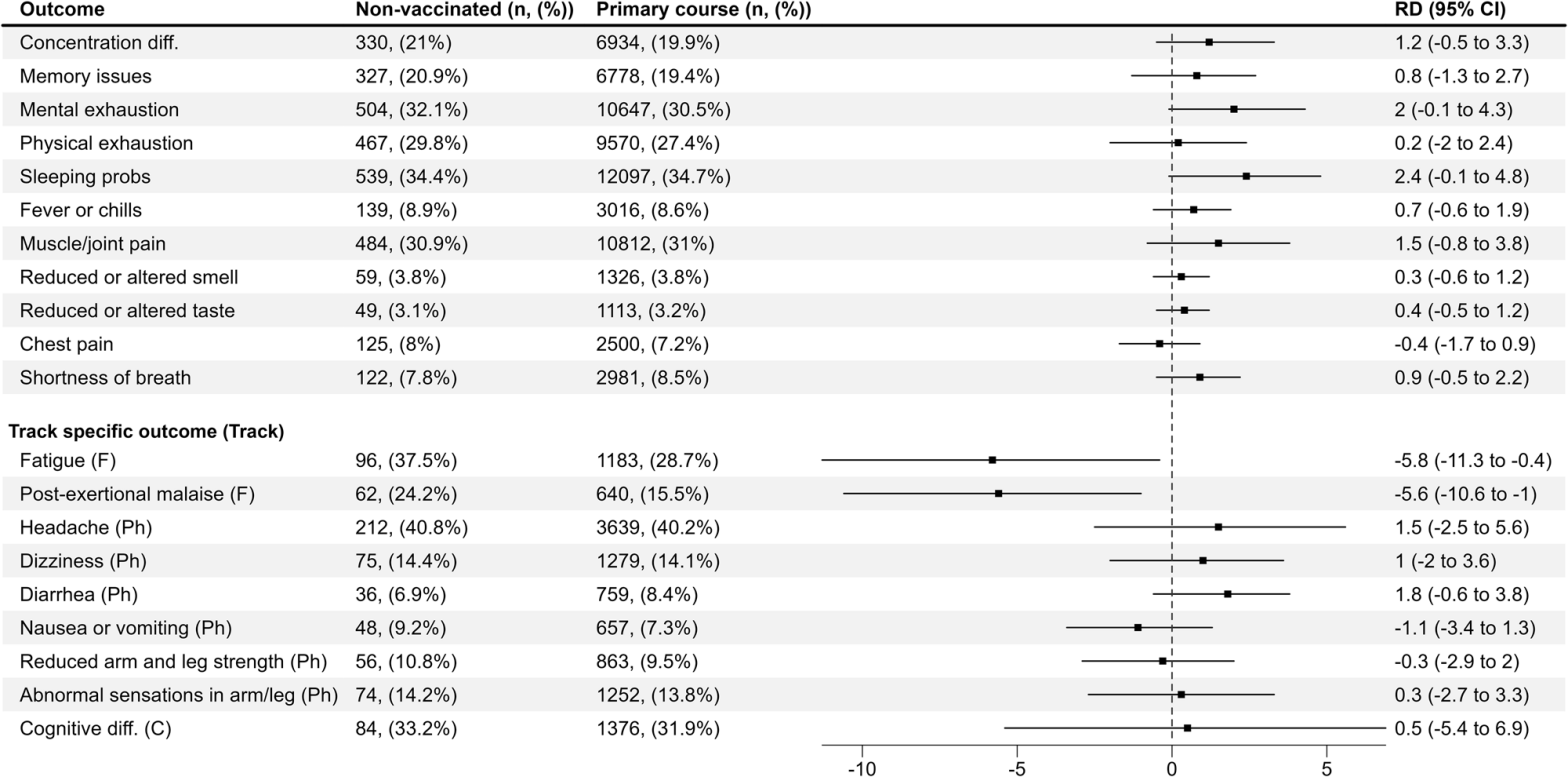
Risk differences (RDs, center) and 95% confidence intervals (CIs, length of error bars) between vaccinated and unvaccinated (ref) participants for self-reported physical, cognitive, and fatigue symptoms. Vaccinated participants refer to individuals who completed their primary course prior to responding to a follow-up questionnaire. Symptoms were self-reported between September 1, 2021 and May 6, 2022. N_unvacinated_ = 1568, N_vaccinated_ = 34,838; Fatigue track (F): based on the Fatigue Assessment Scale and select questions from Depaul Symptom Questionnaire (*N* = 4375, n_unvaccinated_ = 256, n_vaccinated_ = 4119); Physical track (Ph): based on select questions from the 36-Item Short Form Survey Instrument (*N* = 9572, n_unvaccinated_ = 520, n_vaccinated_ = 9052); Cognitive track (C): based on the Cognitive Complaints in Bipolar Disorder Rating Assessment (*N* = 4,568, n_unvaccinated_ = 253, n_vaccinated_ = 4315).

For all general health outcomes, which were answered by all participants regardless of track, there were no significant risk differences between vaccinated and unvaccinated (reference group) individuals. For the track-specific outcomes, we observed significantly higher risk among the unvaccinated individuals compared to vaccinated of both fatigue (RD −5.8%, CI −11.3% to −0.4%) and post-exertional malaise (RD −5.6%, CI −10.6% to −1.0%) (Fig. 1).

### Time since vaccination

**Fig. 2:**
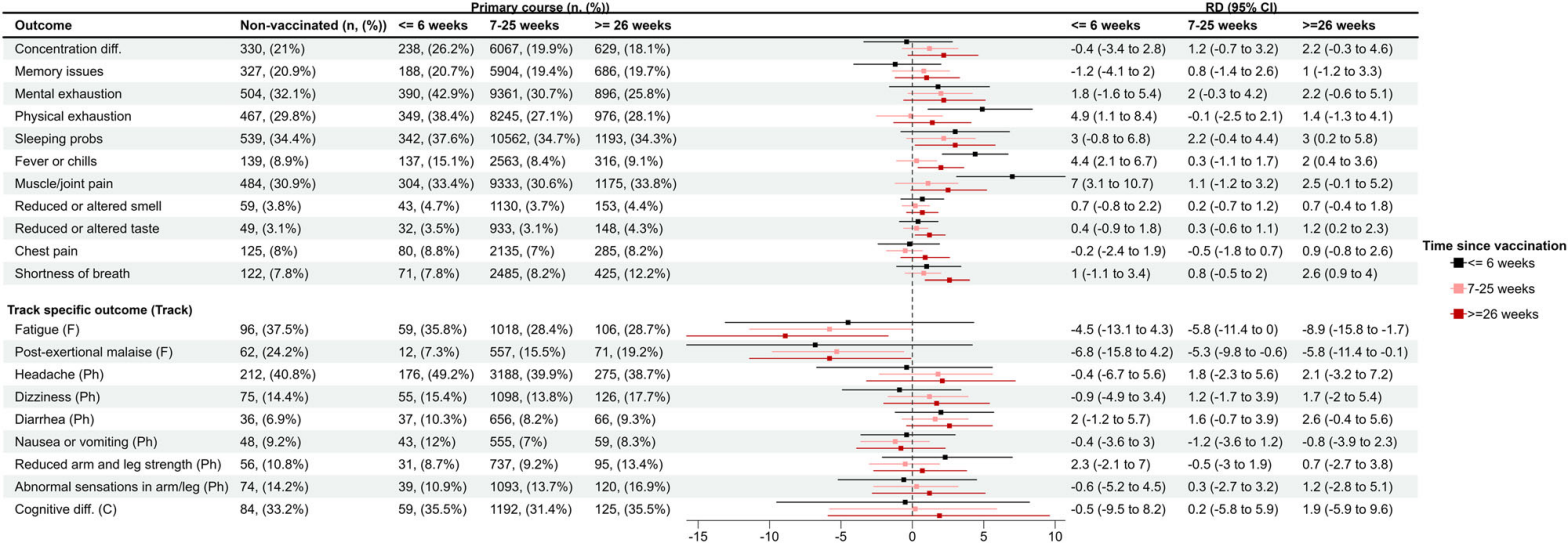
Risk differences (RDs, center) and 95% confidence intervals (CIs, length of error bars) between vaccinated and unvaccinated (ref) participants for self-reported general health problems and general physical symptoms, stratified by time since vaccination. RDs and 95% CIs between vaccinated and unvaccinated for cognitive-, fatigue-related-, and physical symptoms, stratified by time since vaccination. Note that the N persons in each track differs*. Vaccinated participants refer to individuals who completed their primary course prior to responding to a follow-up questionnaire. Symptoms were self-reported between September 1, 2021 and May 6, 2022. *N_unvaccinated_ = 1,568, N_vaccinated_ = 34,838. Fatigue track (F): based on the Fatigue Assessment Scale and select questions from Depaul Symptom Questionnaire (*N* = 4375, n_unvaccinated_ = 256, n_vaccinated_ = 4119). Physical track (Ph): based on select questions from the 36-Item Short Form Survey Instrument (*N* = 9572, n_unvaccinated_ = 520, n_vaccinated_ = 9052). Cognitive track (C): based on the Cognitive Complaints in Bipolar Disorder Rating Assessment (*N* = 4568, n_unvaccinated_ = 253, n_vaccinated_ = 4315).

After stratifying by time since vaccination, we found significant risk differences between vaccinated and unvaccinated persons for physical exhaustion (RD 4.9%, 95% CI 1.1% to 8.4%), fever or chills (RD 4.4%, 95% CI 2.1% to 6.7%), and muscle/joint pain (RD 7.0%, 95% CI 3.1% to 10.7%) within the first six weeks following vaccination (Fig. 2). There was also a significant risk difference between vaccinated and unvaccinated individuals for sleeping problems (RD 3.0, 95% CI 0.2 to 5.8), fever or chills (RD 2.0, 95% CI 0.4 to 3.6), reduced or altered taste (RD 1.2, 95% CI 0.2 to 2.3) and shortness of breath (RD 2.6, 95% CI 0.9 to 4.0) ≥ 26 weeks after vaccination (Fig. 2). Tests of homogeneity were significant at the 0.05 level for physical exhaustion, fever or chills, muscle and joint pain, loss of taste, chest pain, and shortness of breath, suggestive of heterogeneous effects over time.

### Sensitivity analyses

After stratifying by sex, risk differences were only significantly higher among the vaccinated for sleeping problems (RD 3.5%, 95% CI 0.6% to 6.4%) for females and shortness of breath (RD 2.8%, 95% CI 0.7% to 4.9%) for males (Supplementary Fig. 6). In addition, unvaccinated females had a higher risk of post-exertional malaise compared to vaccinated females (RD −9.3%, 95% CI −15.6% to −3.6%). Tests of homogeneity yielded a *p-*value of 0.03 for post-exertional malaise and 0.05 for shortness of breath but otherwise suggested no heterogeneous effects for any other symptoms at a significance level of 0.05. In a sensitivity analyses including only primary course recipients vaccinated with BNT162b2 (Supplementary Fig. 7) and including participants with missing values on possible confounders (Supplementary Fig. 8), we observed no notable differences compared to our main model (Fig. 1). In an analysis restricted to participants who responded before November 15th, 2021 (pre-omicron), risk differences between vaccinated and unvaccinated individuals for sleeping problems, fever or chills, and shortness of breath ≥26 weeks after vaccination were no longer significant (Supplementary Table 7); however, risk differences were higher among the vaccinated than unvaccinated for altered sense of taste ≥26 weeks after vaccination (RD 2.1%, 95% CI 0.7 to 3.4) and muscle/joint pain 7-25 weeks (RD 6.1, 95% CI 1.9 to 10.3) and ≥26 weeks (RD 5.4, 95% CI 0.5 to 10.3) after vaccination.

Poisson models did not show an increase in scores for fatigue or cognition in vaccinated compared to unvaccinated individuals (Supplementary Note 1, Supplementary Fig. 9).

## Discussion

Reassuringly, our results do not provide support for an increased risk of long-term cognitive, fatigue, or physical symptoms following COVID-19 vaccination. The slightly but significantly higher risks for some symptoms (physical exhaustion, fever or chills, and muscle/joint pain) within six weeks of vaccination may be explained by reactogenicity to COVID-19 vaccines, which typically occurs within the first few days of receipt. Shortly after vaccination, such transient side effects are common^13^ and consistent with clinical trial findings^2–5^. The significant differences in risk of symptoms associated with vaccination observed ≥26 weeks after testing may be attributed to undetected SARS-CoV-2 cases, which was supported by a sensitivity analysis of symptoms reported before the surge in Omicron and Delta cases in late autumn 2021. Collectively, these results from a national cohort can help inform on the short- and long-term safety of SARS-CoV-2 primary course vaccination. As SARS-CoV-2 still remains a global health threat^14^, and vaccination has been shown to protect against long covid symptoms^15^, these results may be particularly relevant for individuals who have not received any doses of SARS-CoV-2 vaccines or who did not complete their primary course. The present study may be used to help understand the short- and long-term safety of COVID-19 vaccines. By using self-reported survey data on a broad range of unspecific symptoms related to cognitive difficulties, fatigue, and physical health, this study fills an important gap in COVID-19 vaccine safety research. The extensive national testing strategy in Denmark, in conjunction with register data and a longitudinal survey, allowed us to examine the possible effects COVID-19 vaccination could have on general health among people who were not previously infected with SARS-CoV-2. General symptoms do not always result in individuals seeking medical care, and thus using self-reported symptom data may help capture experiences from more individuals than what could be measured by registered diagnoses. Moreover, given that the EFTER COVID survey does not ask about symptoms in relation to COVID-19 vaccines--but rather symptoms experienced within the past 14 days, this study design reduces concern about self-selection and recall bias. A limitation of this study was the risk for non-response bias among the unvaccinated. At the time of the study, unvaccinated Danish residents comprised less than 10% aged ≥15 years, and thus symptom experiences might not be fully captured within our sample of individuals who chose to participate in the EFTER COVID survey. However, this reflects the reality of studying COVID-19 vaccine safety in countries where the majority of the adult population has completed the primary course. Further participation bias may be of concern, as never having taken a PCR test for SARS-CoV-2 is associated with being unvaccinated in Denmark^16^, and invitations to the survey hinged on testing. We also did not examine symptoms following booster vaccination, as the timing of booster rollout and wide-spread infections with the Omicron variant overlapped during winter 2022. Hence, it was not possible to examine possible long-term symptoms following boosting, as most people who responded to a follow-up questionnaire and reported symptoms would have already been infected with SARS-CoV-2 and could not be included in a study which was designed to examine persons with no history of SARS-CoV-2 infection. We only report on select, pre-defined symptoms. Thus, we cannot draw conclusions about the risk of other symptoms or rare but serious adverse events, which were not inquired about or examined in this study. Due to the EFTER COVID survey design, which invited participants following receipt of negative RT-PCR tests rather than after vaccination, we were also not able to look at risk differences for each symptom at specific points in time. It is also important to acknowledge that our study period (September 1, 2021 to May 5, 2022) encompasses the Omicron wave in Denmark^17^ where, when compared to Delta, rapid spread through immune evasion^18^ and milder cases (i.e., as measured by risk of hospitalization) were observed. This wave implies possible misclassification of some test-negatives, as some participants may have been infected with SARS-CoV-2 but were unaware (e.g., they exhibited subtle or no symptoms), and others may have tested positive using home rapid antigen tests but did not have a registered positive RT-PCR result. To counter this issue, we excluded participants who indicated that they believed they were previously infected with SARS-CoV-2, which would include persons who tested positive at-home. We also conducted a sensitivity analysis restricted to persons who responded prior to the onset of Omicron, where we observed that risk differences between vaccinated and unvaccinated individuals beyond six weeks after vaccination were no longer significant, with the exception of altered sense of taste and muscle/joint pain. These are known symptoms of both acute- and long covid and therefore may be attributable to undetected cases in our study population. This can be further explained by vaccine waning in persons who were vaccinated longer ago before reporting on symptoms during the autumn 2021 to winter 2022 transition, as they would have been more susceptible to SARS-CoV-2 in relation to when they reported their symptoms. Additionally, these individuals may have been less likely to get tested after getting vaccinated and only experiencing mild symptoms following unknown infection. To our knowledge, no other population-based survey studies have investigated such a broad range of unspecific symptoms following COVID-19 vaccination from both a short- (≤6 weeks) and long-term (>6 weeks) perspective. However, a different population-based survey in Denmark, BiCoVac, has documented self-reported adverse reactions in the week following COVID-19 vaccination, where participants were explicitly asked about their symptoms following vaccination. Similar to our study findings, the authors also found that tiredness, muscle pain, general malaise, fever, and joint pain to be fairly prevalent (>5%) in the week following primary course vaccination^19^, although the study did not have a test-negative, unvaccinated control group. A Canadian survey also found that these commonly reported symptoms typically resolved within a week of primary course vaccination^20^. In our study, it is likely that the higher risks observed within the six-week period after vaccination were not consistently present over six weeks; unfortunately, we did not have enough power to examine weekly granularity. Altogether, vaccine safety surveillance systems have allowed countries to identify and relay important safety information about COVID-19 vaccines, as well as inform policy. In the US, early post-authorization safety data enabled the identification of rare but serious adverse events (e.g., anaphylaxis, TTS, myocarditis/pericarditis, Guillain-Barré syndrome), which guided policy changes and updates in vaccines’ emergency use authorization information statements for healthcare providers and recipients^21^. In March 2021, several European countries temporarily suspended the use of ChAdOx1 due to reports of rare but serious thrombosis^22^. In Denmark, rare but serious thrombotic events were also observed shortly after the rollout of Ad26.COV2.S, resulting in the removal of both ChAdOx1 and Ad26.COV2.S from the Danish vaccination program^23,24^. Knowledge and identification of these rare thrombotic events, which are now understood to be TTS cases, continue to progress as investigation of causality and immune-pathogenic mechanisms are on-going^25^. Thus, extraordinary international surveillance efforts and collaboration have enabled countries to flag even very rare safety concerns, which were previously unidentified in the prelicensure trials of COVID-19 vaccines with smaller study populations. Collective knowledge of such rare events should be considered a success of surveillance and promote COVID-19 vaccine confidence. This is particularly important in places with the capacity to vaccinate but where the uptake is still insufficient. Finally, as additional booster doses of COVID-19 vaccines are administered, continuous study and communication of COVID-19 vaccine safety is needed. COVID-19 vaccine safety research has focused on diagnostic endpoints, but many potential issues are not always ascertained well in register data alone. These joint survey- and register-based results may assist in countering vaccine hesitancy rooted in concerns about worsening health after vaccination. This work can be considered together with other findings from post-marketing studies to help inform on the long-term safety of Covid-19 vaccines. In this exploratory, nationwide register and survey study, we found no concerning signs of excess risk of long-term self-reported symptoms among persons aged ≥15 years who were vaccinated against COVID-19. Short-term symptoms which were associated with vaccination confirmed findings from early clinical trials, and other significant findings could be understood in the context of undetected SARS-CoV-2 infection, wide confidence intervals, and multiple testing.

## Methods

### Study context

High vaccine uptake, the timing of mass SARS-CoV-2 testing, and the peak of the Omicron variant were important contextual considerations in the design of this study. Following vaccine rollout in Denmark, more than 90% of Danish residents aged ≥15 years opted to complete their primary course of COVID-19 vaccines. Among the vaccinated, more than 95% received messenger ribonucleic acid (mRNA) vaccines BNT162b2 or MRNA-1273, while fewer received adenovirus vectored vaccines ChAdOx1 and Ad26.COV2.S ( < 5%).

**Fig. 3:**
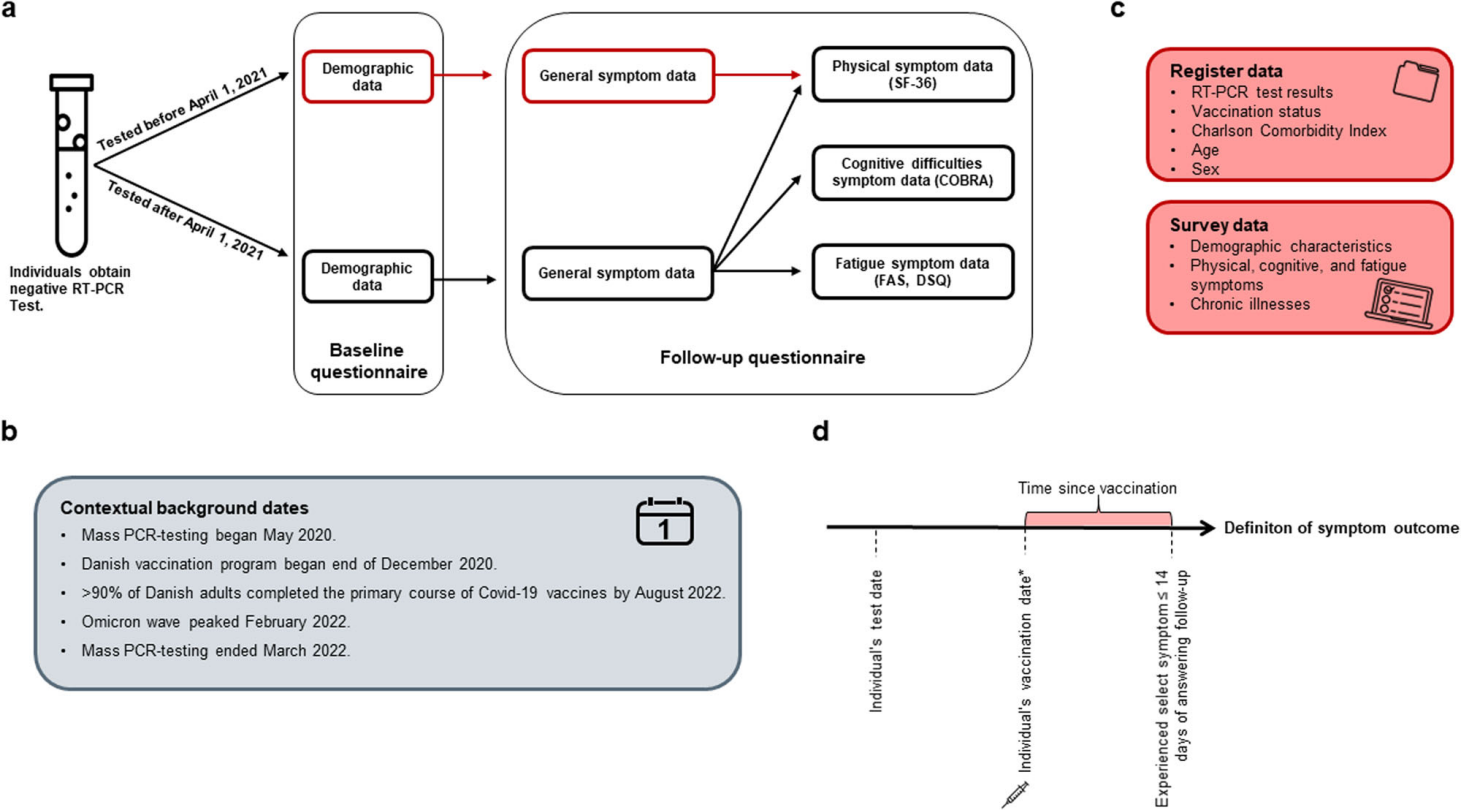
Overview of study design. **a**: Overview of the study design using EFTER-COVID survey data. SF-36: 36-Item Short Form Survey Instrument; COBRA: Cognitive Complaints in Bipolar Disorder Rating Assessment; FAS: Fatigue Assessment Scale; DSQ: DePaul Symptom Questionnaire. **b**: Key contextual background dates inherent to the study design. **c**: Overview of variables pulled from register and survey data. **d**: Definition of symptom outcomes. *If vaccinated. Note that it is also possible for the vaccination date to be prior to the test date.

Denmark’s national SARS-CoV-2 testing strategy provided a unique setting for the conduct of this study, including the possibility to examine a cohort of people with no known history of SARS-CoV-2. Wide-spread reverse transcription polymerase chain reaction (RT-PCR) testing for SARS-CoV-2 began at the end of May 2020 and continued until March 2022, where all test results were registered in the Danish microbiology database (MiBa). Key contextual background dates which influenced our study design are summarized in Fig. 3, Panel B.

### Study design

This nationwide cross-sectional study merged survey and register data. To investigate self-reported symptoms occurring in vaccinated and unvaccinated people, data from the nationwide Danish EFTER-COVID (after Covid) survey were used. The EFTER-COVID survey was initially designed to measure self-reported post-acute symptoms following infection with SARS-CoV-2^12^. Although it was not the primary goal of the survey, the continuous survey component, phrasing of questions, and timely collection of symptom data also yielded an opportunity to examine self-reported symptoms following COVID-19 vaccination.

Invitations to the EFTER COVID survey were initiated by MiBa-registered RT-PCR tests and administered using the national digital mail system, “e-Boks”, which enables secure electronic communication between residents aged ≥15 years and public authorities. Test-negative persons were randomly invited to the survey using incidence density sampling on the test date with a ratio of 2:3 between test-positive and –negative persons. Only test-negative persons who did not receive a positive RT-PCR result prior to responding to the follow-up questionnaire were included in this study. This was done to estimate the absolute effect of SARS-CoV-2 vaccination on long-term symptoms rather than the effect of infection on long-term symptoms.

To reduce participation fatigue and obtain information on a variety of symptoms, participants were placed in different tracks designed to monitor either cognitive, fatigue, or a broader range of symptoms by a ratio of 1:1:2. Participants were asked about these symptoms in relation to the last 14 days from when they responded. The symptoms measured were based on the Cognitive Complaints in Bipolar Disorder Rating Assessment (COBRA), Fatigue Assessment Scale (FAS, © FAS, ild care foundation (www.ildcare.nl)) and select post-exertional malaise (PEM) questions from the DePaul Symptom Questionnaire (DSQ), and select questions from the 36-Item Short Form Survey Instrument (SF-36) (Supplementary Table 2). These tools were chosen due to their validation for measuring for the general health symptoms of interest. Illustration of the EFTER-COVID survey design is available in Fig. 3, Panel A.

### Data sources

An overview of which variables we obtained from survey data and register data is available in Fig. 3, Panel C. EFTER-COVID survey data were collected through Danish- or English-language questionnaires created in SurveyXact^26^, which could be filled out using a PC, tablet, or smartphone. The present study is based on self-reported symptom data collected between September 1, 2021 and May 5, 2022. A baseline questionnaire was used to gather information on height and weight, education, employment, smoking and drinking habits, physical activity, sick leave, and chronic illnesses (diabetes, asthma, high blood pressure, COPD or other chronic lung disease, chronic or frequent headaches/migraines, other), (Supplementary Table 3). In a follow-up questionnaire, all participants were asked about the following: general physical symptoms experienced over the past 14 days (Supplementary Table 1); either cognitive, fatigue, or additional physical symptoms experienced over the last 14 days dependent on the randomly assigned track (Supplementary Table 2), and pre-existing conditions (Supplementary Table 3).

Individual level data from all data sources were linked using the unique identifier (the CPR-number) from the Danish Civil Registration System. Using the CPR-number, the EFTER COVID survey data were enriched with information on age and sex from the Danish Civil Registration System, information on COVID-19 vaccinations from the Danish Vaccination Register (DDV), as well as information on comorbidities from the Danish National Patient Register (DNPR) five years prior to each participant’s test date. From the DNPR, we obtained information on in- and outpatient diagnoses coded using the 10^th^ revision of the International Statistical Classification of Diseases and Related Problems (ICD-10), which enabled the calculation of Charlson Comorbidity Index scores. SARS-CoV-2 test results were retrieved from MiBa.

### Exposure

Participants were considered “vaccinated” (exposed group) if they had completed the primary course and “unvaccinated” (reference group) if they received no doses of Covid-19 vaccines. Completion of the primary course was defined as having received two doses of BNT162b2/MRNA-1273/ChAdOx1, a combination of these, or a single dose of Ad26.COV2.S by the time each individual responded to their follow-up questionnaire. Each individual’s vaccination status and time since completing the primary course of vaccination were defined according to the response date to the follow-up questionnaire. Receipt of the primary course is based on the date of receiving the second dose (or for a single dose of Ad26.COV2.S).

### Exclusion criteria

Apart from excluding test-positives for SARS-CoV-2, we excluded individuals who believed they previously had SARS-CoV-2. This included individuals who self-reported that they had previously obtained a seropositive result for SARS-CoV-2 or tested positive on a home rapid antigen test. Furthermore, we did not include individuals who had received incomplete vaccination (e.g., a single dose of an mRNA vaccine) or booster doses prior to responding to the follow-up questionnaire. We were not able to examine incomplete vaccination because the vast majority (92%) completed the primary course within six weeks after the first dose, and few participants reported on symptoms within the period after their first dose. We also could not examine booster doses, as boosting occurred at the same time as widespread omicron infections. Receipt of a booster dose is based on the date of receipt and defined as a third dose of BNT162b2/MRNA-1273 or a second dose of Ad26.COV2.S. In addition, individuals with missing values on confounding variables (e.g., height, weight, smoking-, and drinking habits) were excluded. Further information on our inclusion and exclusion criteria is available in Supplementary Fig. 1.

### Outcomes

General health outcomes, which were reported regardless of track, included fever or chills, concentration difficulties, memory issues, mental exhaustion, physical exhaustion, sleeping problems, muscle/joint pain, reduced or altered smell, reduced or altered taste, chest pain, and shortness of breath. Track-specific outcomes included general cognitive difficulties (cognitive track), general fatigue and post-exertional malaise (fatigue track), as well as headache, dizziness, diarrhea, nausea or vomiting, reduced arm and leg strength, sleeping or tingling sensation or other abnormal sensations in the legs (physical track). All outcomes were reportedly experienced within 14 days prior to responding to the follow-up questionnaire. We then considered a symptom to be a possible “long-term” side effect of vaccination if experienced >6 weeks after SARS-CoV-2 vaccination, which we determined by using the follow-up questionnaire response date and the registered vaccination date.

The cognitive and fatigue outcomes were based on multiple questions and single binary outcomes were defined using standardized scores for fatigue and post-exertional malaise, which were derived from the FAS and DSQ, respectively. For the cognitive outcome, we used a cut-off value equal to 8.56 between case (cognitive difficulties) and no case (no cognitive difficulties). This value was based on the mean COBRA score of 68 healthy controls in a Danish study from 2015^27^. A graphic showing how we defined symptom outcomes is available in Fig. 3, Panel D.

### Statistical methods

The prevalence cognitive, fatigue, and physical symptoms (all coded as binary variables) among unvaccinated and vaccinated individuals were compared using risk differences (RDs). *P*-values for the association between vaccination status and the survey participant characteristics were estimated using student’s *t*-test for continuous variables and Pearson’s Chi-squared test for categorical variables.

We used parametric g-computation on logistic regression^28^ to estimate RDs with 95% confidence intervals obtained using bootstrap random resampling with 1000 iterations comparing vaccinated and unvaccinated individuals with adjustments for age, sex, obesity, smoking and alcohol habits, self-reported comorbidities, and Charlson Comorbidity Index score. These adjustments were decided upon based on correlations with vaccination status and symptom outcomes. Further information on potential confounder variables is available in Supplementary Materials (Supplementary Table 3).

For each individual, we defined time since vaccination as the time from vaccination to their response to the follow-up questionnaire. Time since vaccination was then categorized as ≤6 weeks, 7–25 weeks, and ≥26 weeks (Supplementary Table 4). The six-week cut-off point was chosen to capture the acute phase for physical symptoms (e.g., the first week after receipt of the primary course), but also to explore fatigue, and cognitive symptoms while the immune system responds to the antigen and for some time after. The 26-week cut-off point was selected due to the timing of the rollout of vaccines in Denmark in relation to the timing of the survey and subsequent number of responses. For each outcome, RDs stratified on time since vaccination were computed. In addition, we obtained RDs for each outcome stratified on sex.

To investigate whether there is homogeneity of effects across the categories of the stratification variables, we carried out likelihood ratio tests between the main underlying logistic regression model and (1) a logistic regression model with the aforementioned possible confounders and vaccination status corresponding to the categories of time since vaccination and (2) a logistic regression model with the aforementioned possible confounders and an interaction term between sex and vaccination status. Moreover, we conducted three sensitivity analyses: (1) restricting primary course recipients only to those who received two doses of BNT162b2, (2) including participants with missing values on possible confounding variables, where missingness was treated as a separate category in each variable, and (3) restricting responses to only those before November 15^th^, 2021 (pre-Omicron and large surge of autumn Delta cases).

We further explored the relationship between vaccination status and fatigue and cognitive scores by fitting Poisson regression models (for details see Supplementary Note 1, Supplementary Fig. 5).

All statistical analyses were carried out in R version 4.1.3^29^. The R-package “riskCommunicator”^28^ was used for modelling and “forestploter”^30^ for data visualization (forest plots).

### Reporting summary

Further information on research design is available in the Nature Research Reporting Summary linked to this article.

### Supplementary information

Supplemental Material

Reporting Summary

## Data Availability

The data are becoming- or are already available for research upon reasonable request to Statens Serum Institut and within the framework of the Danish data protection legislation and any required permission from relevant authorities. EO affirms that the manuscript is an honest, accurate, and transparent account of the present study; that no important aspects of the study have been omitted; and that any discrepancies from the study have been planned and explained.

## Code availability

The underlying code for this study is not publicly available.
