# Supplementary material for: A register and questionnaire study of long-term general health symptoms following SARS-CoV-2 vaccination in Denmark
Source: NPJ Vaccines. 2024 Mar 4;9:52. doi: 10.1038/s41541-024-00844-w (PMC10912726; doi:10.1038/s41541-024-00844-w)
Supplement: Supplementary file 1 — Supplemental Material [file 41541_2024_844_MOESM1_ESM.docx]

**Supplementary Material**

Table of contents

[Supplementary Figure 1 Flowchart of study population 2](#_Toc158883980)

[Supplementary Table 1 Overview of general health symptoms (study outcomes). 3](#_Toc158883981)

[Supplementary Table 2 Overview of track-specific symptoms (study outcomes). 4](#_Toc158883982)

[Supplementary Table 3 Overview of confounder variables. 5](#_Toc158883983)

[Supplementary Table 4 Overview of stratification variables. 6](#_Toc158883984)

[Supplementary Table 5 Proportions of primary course recipients and unvaccinated individuals in each EFTER COVID track. Total study population N=36,436 (n_vaccinated_ = 34,868, n_unvaccinated_ = 1,568). 7](#_Toc158883985)

[Supplementary Figure 2 Distribution of responses by time passed since completion of the primary course and type(s) of vaccine received. 8](file:///H:\SFSD\EPIFORSK\EIOR\Efter_Covid\vaccine_safety\latest\20230727\Response_reviewers_npjvaccine\20240212\supplementary_materials_20240215.docx#_Toc158883986)

[Supplementary Figure 3 Participation by vaccination overview. 9](#_Toc158883987)

[Supplementary Figure 4 Survey response dates divided on time since vaccination (≤6 weeks, 7-25 weeks, ≥26 weeks). 10](#_Toc158883988)

[Supplementary Table 6 Characteristics by survey completion. 11](#_Toc158883989)

[Supplementary Figure 5 Prevalence plots for symptoms by age group and sex. 12](#_Toc158883990)

[Supplementary Figure 6 Risk differences (RDs) and 95% confidence intervals (CI) between vaccinated (primary course) and unvaccinated (ref) participants for self-reported cognitive-, fatigue-related, and physical symptoms, stratified by sex. 13](file:///H:\SFSD\EPIFORSK\EIOR\Efter_Covid\vaccine_safety\latest\20230727\Response_reviewers_npjvaccine\20240212\supplementary_materials_20240215.docx#_Toc158883991)

[Supplementary Figure 7 Sensitivity analysis with risk differences (RDs) and 95% confidence intervals (CI) between primary course recipients of BNT162b2 and unvaccinated (ref) participants for self-reported cognitive-, fatigue-related, and physical symptoms. 14](file:///H:\SFSD\EPIFORSK\EIOR\Efter_Covid\vaccine_safety\latest\20230727\Response_reviewers_npjvaccine\20240212\supplementary_materials_20240215.docx#_Toc158883992)

[Supplementary Figure 8 Risk differences (RDs) and 95% confidence intervals (CIs) between 39,562 vaccinated and 1,792 unvaccinated (ref) participants* for self-reported cognitive-, fatigue-related-, and physical symptoms. 15](#_Toc158883993)

[Supplementary Table 7 Risk differences (RDs) and 95% confidence intervals (CIs) between 16,102 vaccinated and 430 unvaccinated (ref) participants* for self-reported general health symptoms. 16](#_Toc158883994)

[Supplementary Figure 9: Distribution of fatigue and cognition scores. 17](#_Toc158883995)

[Supplementary Note 1 Description of Poisson Regression Models 18](#_Toc158883996)

# *
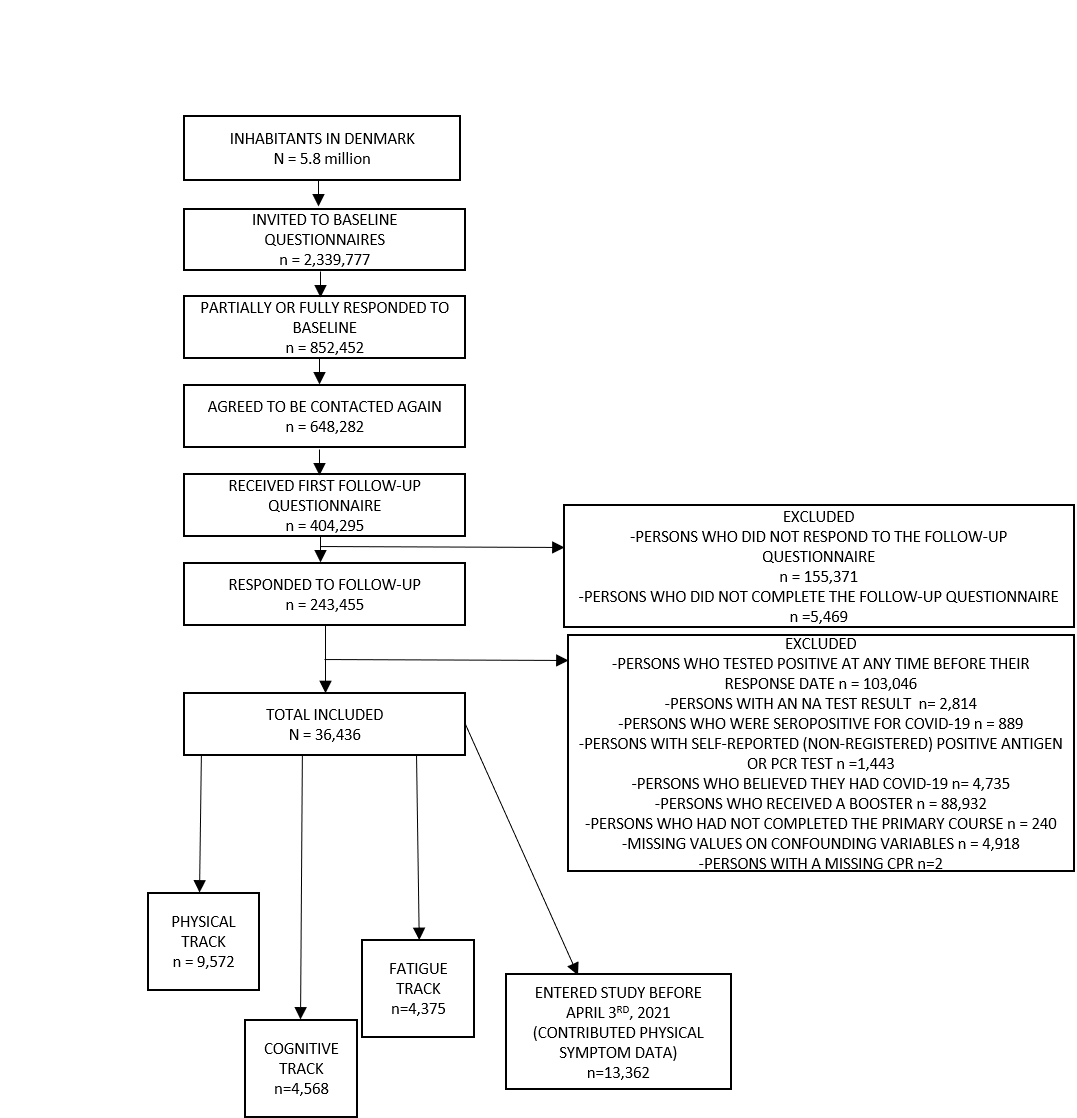
***Supplementary Figure 1** Flowchart of study population

Of the 2,339,777 individuals invited to participate in the EFTER COVID baseline questionnaire, 852,452 responded by May 6, 2022 (response rate for the baseline questionnaire = 36.4%). Of the 404,295 individuals who agreed to be contacted after completing a baseline questionnaire and received a follow-up questionnaire, 243,455 responded (response rate for the follow-up questionnaire = 60.2%) by May 6, 2022.

| **Supplementary Table 1** Overview of general health symptoms (study outcomes). | | | | |
| --- | --- | --- | --- | --- |
| **Name** | **Description** | **Data type** | **Categorization** | **Data source** |
| Difficulties concentrating | Self-reported. Experienced in the past 14 days. | Categorical variable. | “Yes”  “No” | EFTER-COVID questionnaires. |
| Issues with memory | Self-reported. Experienced in the past 14 days. | Categorical variable. | “Yes”  “No” | EFTER-COVID questionnaires. |
| Mental exhaustion | Self-reported. Experienced in the past 14 days. | Categorical variable. | “Yes”  “No” | EFTER-COVID questionnaires. |
| Physical exhaustion | Self-reported. Experienced in the past 14 days. | Categorical variable. | “Yes”  “No” | EFTER-COVID questionnaires. |
| Sleep problems | Self-reported. Experienced in the past 14 days. | Categorical variable. | “Yes”  ‘No’ | EFTER-COVID questionnaires. |
| Shortness of breath | Self-reported. Experienced in the past 14 days. | Categorical variable. | “Yes”  “No” | EFTER-COVID questionnaires. |
| Fever or chills | Self-reported. Experienced in the past 14 days. | Categorical variable. | “Yes”  “No” | EFTER-COVID questionnaires. |
| Reduced or altered sense of taste | Self-reported. Experienced in the past 14 days. | Categorical variable. | “Yes”  “No” | EFTER-COVID questionnaires. |
| Reduced or altered sense of smell | Self-reported. Experienced in the past 14 days. | Categorical variable. | “Yes”  “No” | EFTER-COVID questionnaires. |
| Chest pain | Self-reported. Experienced in the past 14 days. | Categorical variable. | “Yes”  “No” | EFTER-COVID questionnaires. |
| Muscle or joint pain | Self-reported. Experienced in the past 14 days. | Categorical variable. | “Yes”  “No” | EFTER-COVID questionnaires. |

| **Supplementary Table 2** Overview of track-specific symptoms (study outcomes). | | | | |  |
| --- | --- | --- | --- | --- | --- |
|  | **Name** | **Description** | **Data type** | **Categorization** | **Data Source** |
| **Physical** | Headache | Self-reported. Experienced in the past 14 days. Based on SF-36. | Categorical variable. | Yes  No | Physical track EFTER-COVID questionnaires. |
|  | Diarrhea | Self-reported. Experienced in the past 14 days. Based on SF-36. | Categorical variable. | Yes  No | Physical track EFTER-COVID questionnaires. |
|  | Dizziness | Self-reported. Experienced in the past 14 days. Based on SF-36. | Categorical variable. | Yes  No | Physical track EFTER-COVID questionnaires. |
|  | Nausea or vomiting | Self-reported. Experienced in the past 14 days. Based on SF-36. | Categorical variable. | Yes  No | Physical track EFTER-COVID questionnaires. |
|  | Reduced arm and leg strength | Self-reported. Experienced in the past 14 days. Based on SF-36. | Categorical variable. | Yes  No | Physical track EFTER-COVID questionnaires. |
|  | Sleeping or tingling or other abnormal sensations in legs | Self-reported. Experienced in the past 14 days. Based on SF-36. | Categorical variable. | Yes  No | Physical track EFTER-COVID questionnaires. |
| **Cognitive** | Cognitive difficulties | Based on questions from the COBRA. | Categorical variable | No case (≤ 8.56 quantile)  Case (>8.56 quantile) | Cognitive track EFTER-COVID questionnaires. |
| **Fatigue** | Fatigue | Based on questions from the FAS. | Categorical variable. | No fatigue (10-21)  Substantial fatigue (22-50) | Fatigue track EFTER-COVID questionnaires. |
|  | Post-exertional Malaise | Based on questions about post-exertional malaise (PEM) from the DSQ. | Categorical variable. | Indicative of PEM (A frequency AND severity score of at least 2 AND a 2 on any items 1-5)  Not indicative of PEM | Fatigue track EFTER-COVID questionnaires. |
| SF-36: 36-Item Short Form Survey Instrument, COBRA: Cognitive Complaints in Bipolar Disorder Rating Assessment , FAS: Fatigue Assessment Scale, DSQ: DePaul Symptom Questionnaire.  *The FAS, DSQ, and SF-36 were selected due to each tool being well-established and validated for measuring fatigue, post-exertional malaise, and physical symptoms, respectively. The COBRA was selected to investigate subjective cognitive difficulties. COBRA measures executive function, processing speed, working memory, verbal learning and memory, attention/concentration and mental tracking. | | | | | |

| **Supplementary Table 3** Overview of confounder variables. | | | |  |
| --- | --- | --- | --- | --- |
| **Name** | **Description** | **Data type** | **Categorization** | **Data source** |
| Age | Age group (age on day of test). | Categorical variable. | 15-19 years  20-29 years  30-39 years  40-49 years  50-59 years  60-69 years  ≥70 years | Danish Civil Registration System |
| Sex | Biological sex. | Categorical variable. | Female  Male | Danish Civil Registration System |
| Charlson Comorbidity Index Score | Based on the Charlson Comorbidity Index. | Categorical variable. | 0  1  2  ≥3 | Danish Civil Registration System |
| Self-reported pre-existing conditions | Diabetes, asthma, high blood pressure, COPD or other chronic lung disease, chronic or frequent headaches (incl. migraines), other chronic disease. | Categorical variable. | Yes  No | EFTER-COVID questionnaires |
| Obesity | Based on the Body Mass Index (BMI). Defined as BMI ≥ 30kg/m^2^ for individuals aged ≥18 years. For individuals aged 15-17 years, international cutoff points for obesity by sex and age were used (Cole et al.)*. | Categorical variable. | Not obese  Obese | EFTER-COVID questionnaires |
| Smoking | Self-reported smoking habits. | Categorical variable | Never  Not in past five years  Yes in past five years  Daily, less than 10  Daily, more than 10  Electronic cigarette | EFTER-COVID questionnaires. |
| Alcohol | Self-reported drinking habits. One drink corresponds to approximately 15 ml of pure alcohol. | Categorical variable | Never (0 drinks)  Moderate (1-10 drinks per week)  Heavy (10+ drinks per week) | EFTER-COVID questionnaires. |
| *Cole TJ, Bellizzi MC, Flegal KM, Dietz WH. Establishing a standard definition for child overweight and obesity worldwide: international survey. Bmj. 2000;320(7244):1240. | | | | |

| **Supplementary Table 4** Overview of stratification variables. | | | | |
| --- | --- | --- | --- | --- |
| **Name** | **Description** | **Data type** | **Categorization** | **Data Source** |
| Time since vaccination* | Difference in days between answer date and vaccination date. All unvaccinated individuals are included in each strata. | Categorical variable. | ≤6 weeks  7 – 25 weeks  ≥26 weeks | Danish Vaccination Register |
| Sex | Biological sex | Categorical | Male  Female | Danish Civil Registration System |
| *The six-week cut-off point was chosen to capture the acute phase for physical symptoms (e.g., the first week after receipt of the primary course), but also to explore fatigue, and cognitive symptoms while the immune system responds to the antigen and for some time after. The 26-week cut-off point was selected due to the timing of the rollout of vaccines in Denmark in relation to the timing of the survey and subsequent number of responses. | | | | |

| **Supplementary Table 5** Proportions of primary course recipients and unvaccinated individuals in each EFTER COVID track. Total study population N=36,436 (n_vaccinated_ = 34,868, n_unvaccinated_ = 1,568). | | | | |
| --- | --- | --- | --- | --- |
|  | **Cognitive** | **Fatigue** | **Physical** | **Answered before April 3, 2021 (physical symptoms)** |
|  | (N=4,568) | (N=4,375) | (N=9,572) | (N=13,362) |
| **Vaccination Status** |  |  |  |  |
| Primary Course | 4,315 (94.5%) | 4,119 (94.1%) | 9,052 (94.6%) | 13,079 (97.9%) |
| Unvaccinated | 253 (5.5%) | 256 (5.9%) | 520 (5.4%) | 283 (2.1%) |

# **Supplementary Figure 2** Distribution of responses by time passed since completion of the primary course and type(s) of vaccine received.


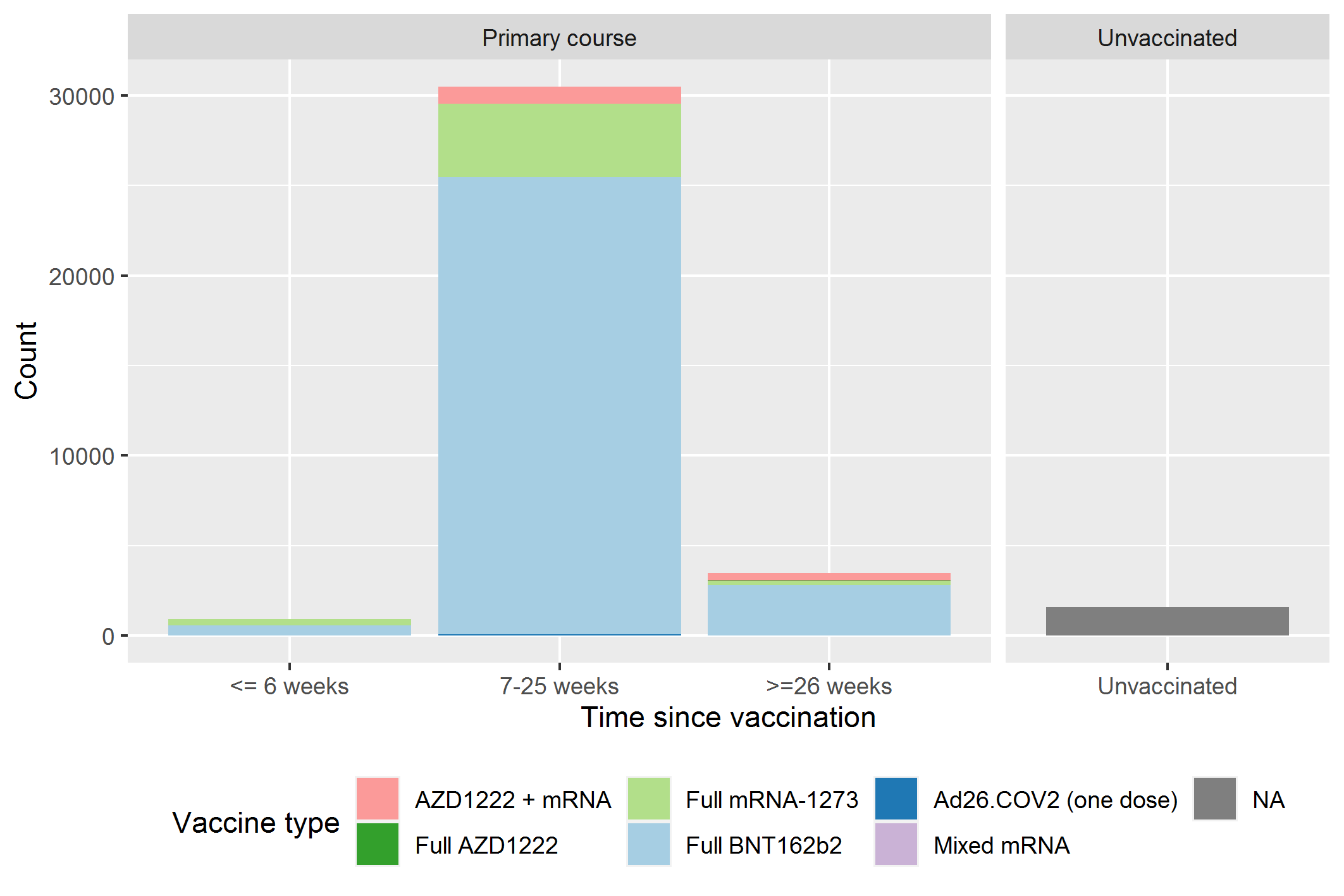


Total study population N=36,436 (n_vaccinated_ = 34,868, n_unvaccinated_ = 1,568).
mRNA: messenger ribonucleic acid

NA: not available (no vaccine received)

**
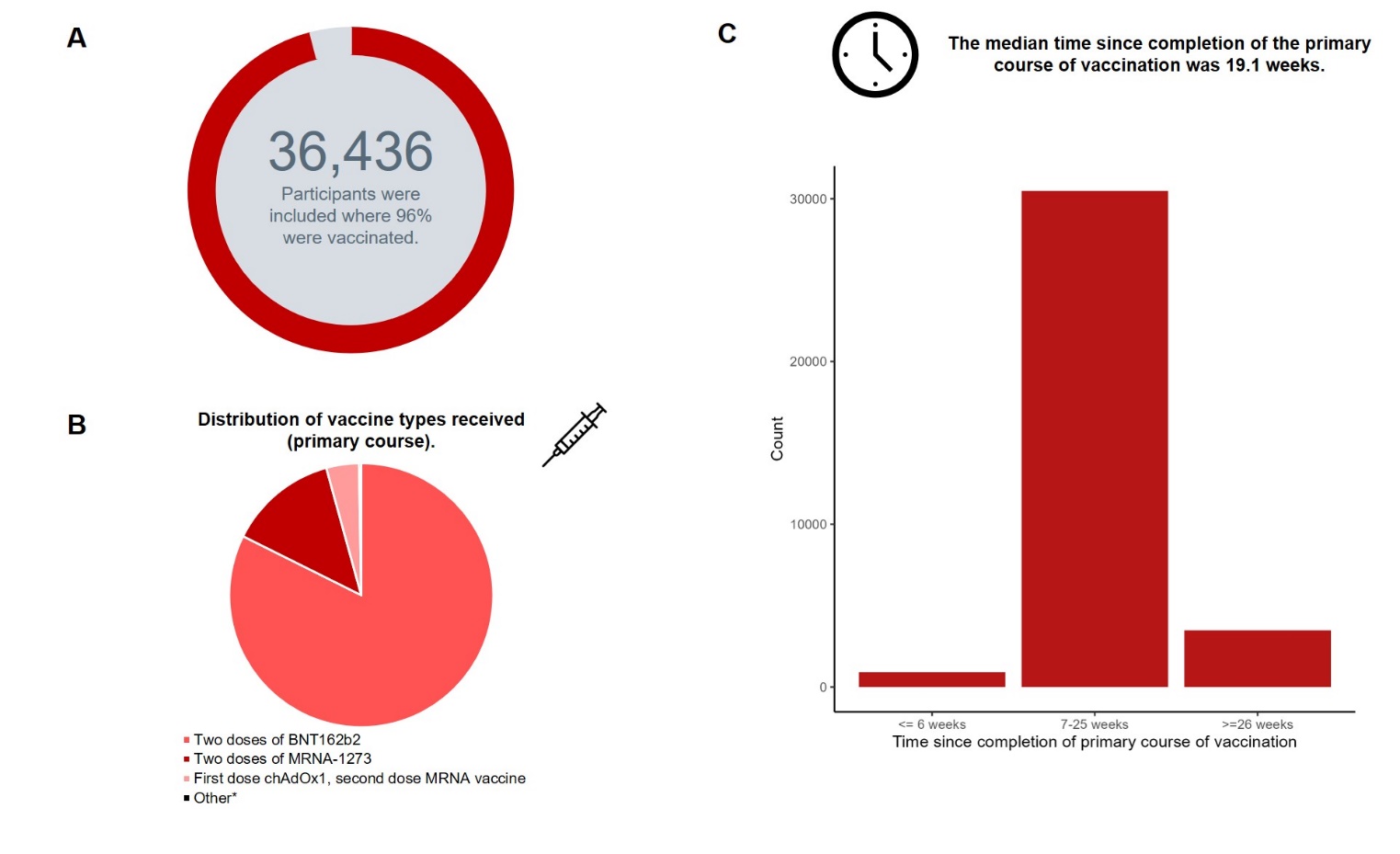
****Supplementary Figure 3** Participation by vaccination overview. Panel A: Number of participants included and response rate. Panel B: Distribution of vaccines received (primary course). Completion of the primary course was defined as having received two doses of BNT162b2/MRNA-1273/ChAdOx1, a combination of these, or a single dose of Ad26.COV2.S by the time each individual responded to their follow-up questionnaire. Each individual’s vaccination status and time since completing the primary course of vaccination were defined according to the response date to the follow-up questionnaire. Receipt of the primary course is based on the date of receiving the second dose (or for a single dose of Ad26.COV2.S). Panel C: N survey participants by time since vaccination categories (≤6 weeks, 7-25 weeks, ≥26 weeks). Time since vaccination refers to the time passed between reporting on symptoms experienced in the last 14 days and the completion date of the primary course.

# **Supplementary Figure 4** Survey response dates divided on time since vaccination (≤6 weeks, 7-25 weeks, ≥26 weeks).


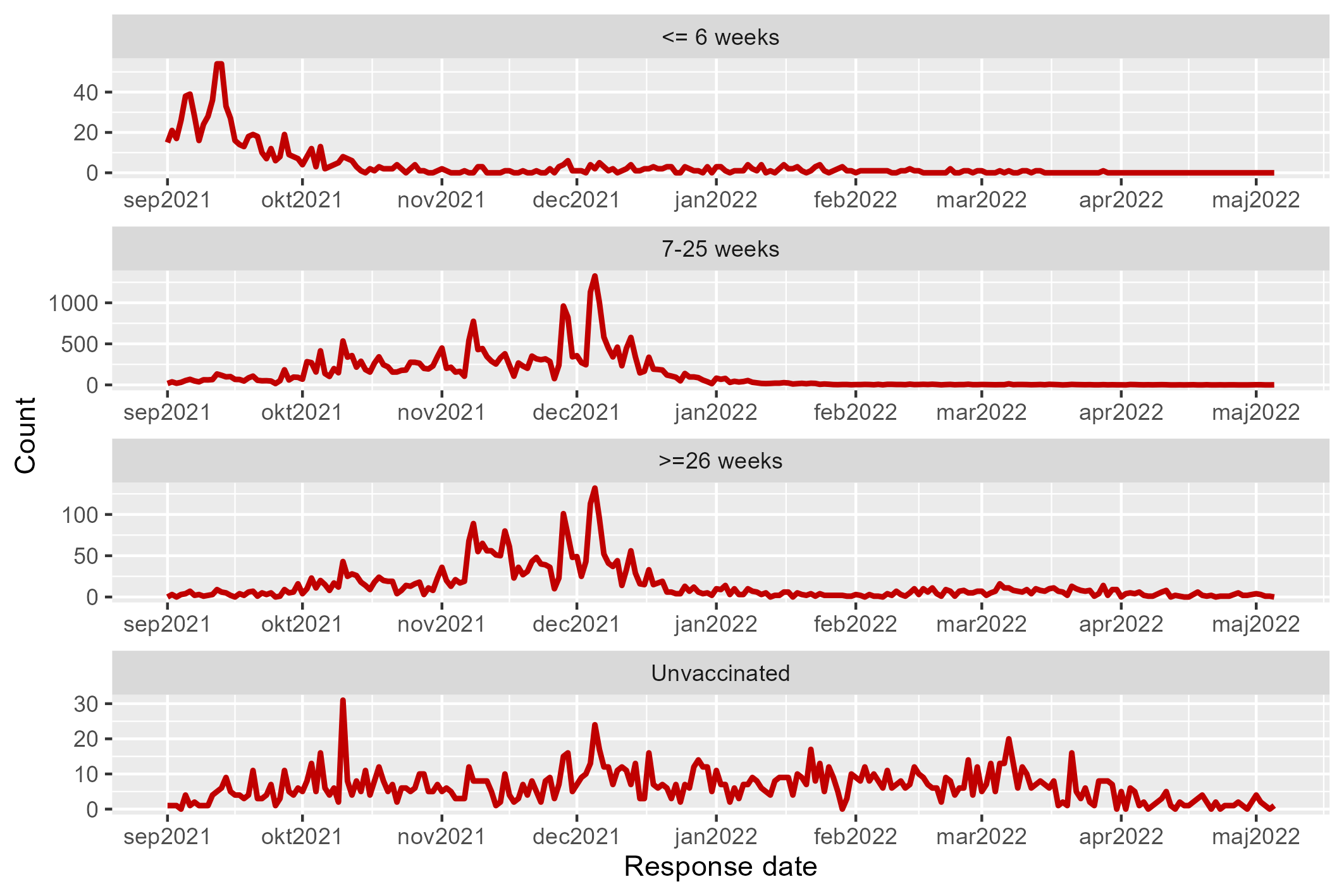


| **Supplementary Table 6** Characteristics by survey completion. | | | | |
| --- | --- | --- | --- | --- |
|  | **Fully complete** | **Incomplete** | **Partially complete** | **Overall** |
|  | (N=243,455) | (N=155,371) | (N=5,469) | (N=404,295) |
| **Sex** |  |  |  |  |
| Female | 148409 (61.0%) | 93130 (59.9%) | 3652 (66.8%) | 245191 (60.6%) |
| Male | ≤95049 (39.0%) | ≤62245 (40.1%) | ≤1822 (33.2%) | ≤159106 (39.4%) |
| Missing | ≤5 (0.0%) | ≤5 (0.0%) | ≤5 (0%) | ≤5 (0.0%) |
| **Age category** |  |  |  |  |
| 15-19 | 4322 (1.8%) | 7204 (4.6%) | 358 (6.5%) | 11884 (2.9%) |
| 20-29 | 15678 (6.4%) | 21319 (13.7%) | 735 (13.4%) | 37732 (9.3%) |
| 30-39 | 20656 (8.5%) | 22989 (14.8%) | 695 (12.7%) | 44340 (11.0%) |
| 40-49 | 38314 (15.7%) | 31008 (20.0%) | 935 (17.1%) | 70257 (17.4%) |
| 50-59 | 63811 (26.2%) | 36294 (23.4%) | 1221 (22.3%) | 101326 (25.1%) |
| 60-69 | 60260 (24.8%) | 23563 (15.2%) | 895 (16.4%) | 84718 (21.0%) |
| 70+ | ≤40417 (16.6%) | ≤12998 (8.4%) | ≤635 (11.5%) | ≤54040 (13.4%) |
| Missing | ≤5 (0.0%) | ≤5 (0.0%) | ≤5 (0%) | ≤5 (0.0%) |
| **Charlson Comorbidity Index** |  |  |  |  |
| 0 | 205990 (84.6%) | 136959 (88.1%) | 4669 (85.4%) | 347618 (86.0%) |
| 1 | 17825 (7.3%) | 9484 (6.1%) | 406 (7.4%) | 27715 (6.9%) |
| 2 | 14307 (5.9%) | 6421 (4.1%) | 272 (5.0%) | 21000 (5.2%) |
| 3 or more | ≤5336 (2.2%) | ≤2511 (1.6%) | ≤127 (2.2%) | ≤7964 (2.0%) |
| Missing | ≤5 (0.0%) | ≤5(0.0%) | ≤5 (0%) | ≤5 (0.0%) |
| **Test result** |  |  |  |  |
| Negative | 137595 (56.5%) | 80865 (52.0%) | 3308 (60.5%) | 221768 (54.9%) |
| positive | 103046 (42.3%) | 71615 (46.1%) | 2100 (38.4%) | 176761 (43.7%) |
| Missing | 2814 (1.2%) | 2891 (1.9%) | 61 (1.1%) | 5766 (1.4%) |

**
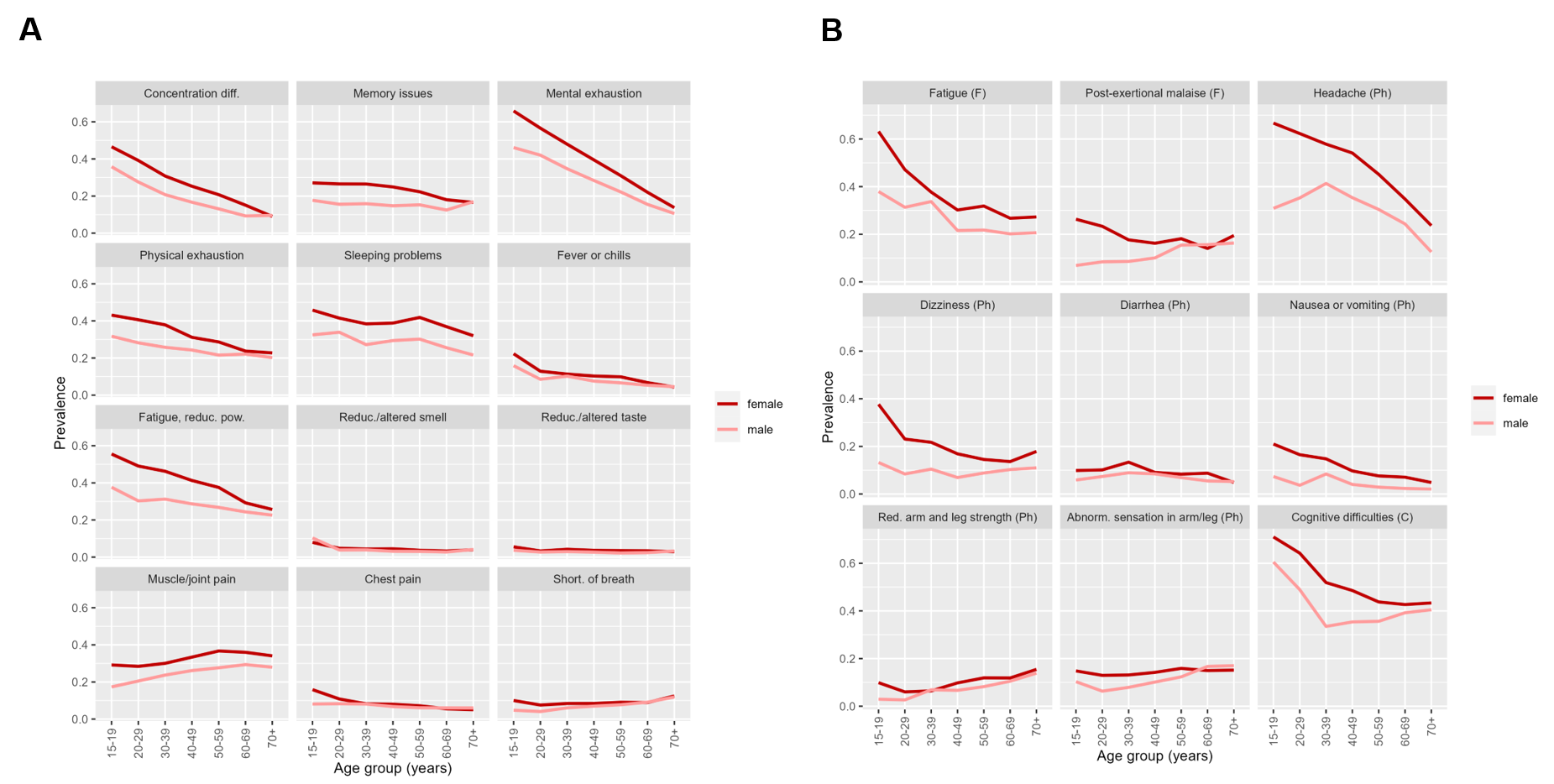
****Supplementary Figure 5** Prevalence plots for symptoms by age group and sex. Prevalence of self-reported general health symptoms (Panel A) and track-specific symptoms (Panel B) among vaccinated and unvaccinated EFTER COVID survey participants by age group and sex. All participants had never received a positive RT-PCR result for SARS-CoV-2 prior to reporting symptoms. Symptoms were self-reported between September 1, 2021 and May 6, 2022.

Cognitive track (C): based on the Cognitive Complaints in Bipolar Disorder Rating Assessment (N=4,568); Fatigue track (F): based on the Fatigue Assessment Scale and select questions from the Depaul Symptom Questionnaire (N=4,375); Physical track (Ph): based on select questions from the 36-Item Short Form Survey Instrument (N=9,572)

**Supplementary Figure 6** Risk differences (RDs) and 95% confidence intervals (CI) between vaccinated (primary course) and unvaccinated (ref) participants for self-reported cognitive-, fatigue-related, and physical symptoms, stratified by sex. Vaccinated refers to individuals who completed the primary course prior to responding to a follow-up questionnaire.

Total study population N=36,436 (n_vaccinated_ = 34,868, n_unvaccinated_ = 1,568).

N_female_ = 21,563 (n_vaccinated_ = 20,449 , n_unvaccinated_ = 1,114), N_male_ = 14,873 (n_vaccinated =_ 14,419_,_ n_unvaccinated_ = 454).

Fatigue track (F): based on the Fatigue Assessment Scale and select questions from Depaul Symptom Questionnaire (N=4,375, n_unvaccinated_ = 256, n_vaccinated_ = 4,119)

Physical track (Ph): based on select questions from the 36-Item Short Form Survey Instrument (N=9,572, n_unvaccinated_ = 520, n_vaccinated_ = 9,052)

Cognitive track (C): based on the Cognitive Complaints in Bipolar Disorder Rating Assessment (N=4,568, n_unvaccinated_ = 253, n_vaccinated_ = 4,315)

*
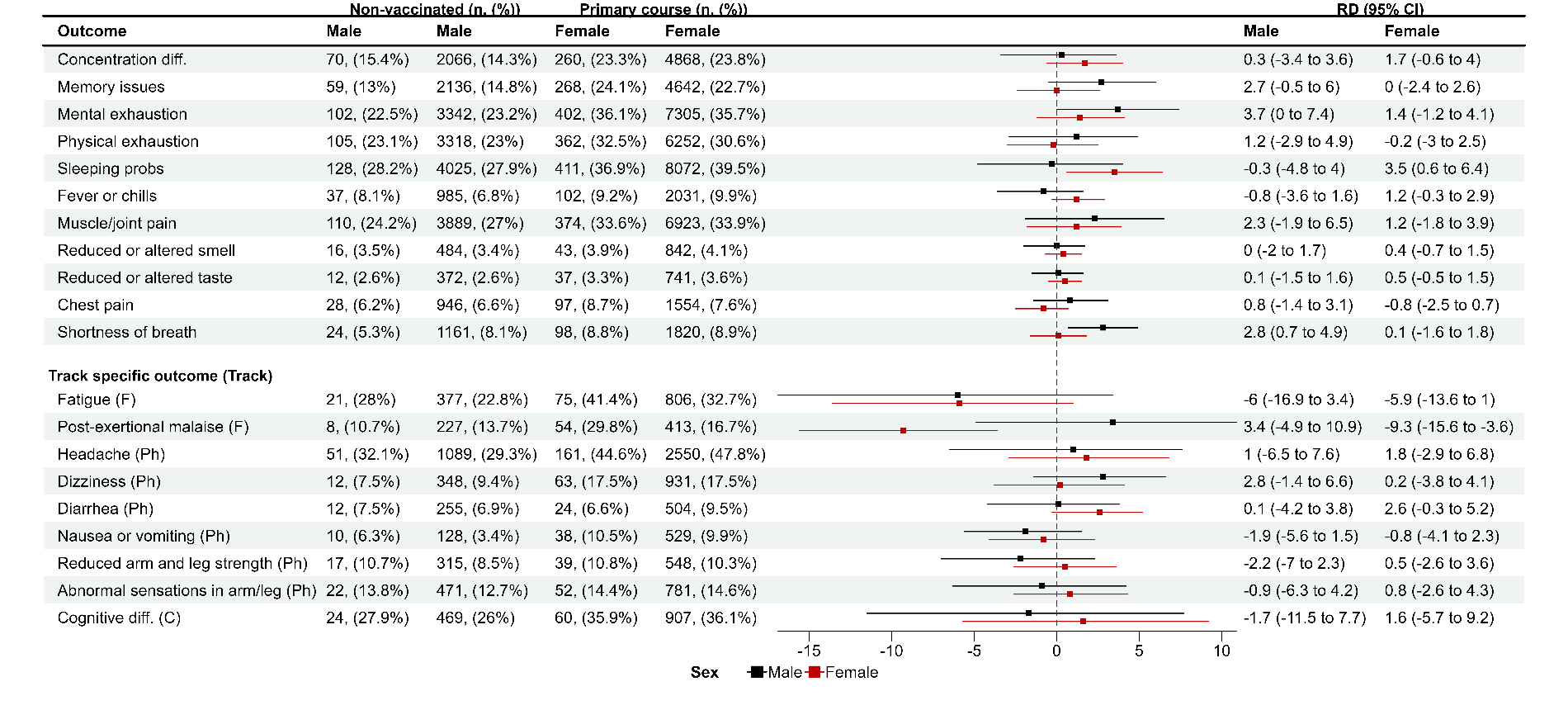
*

*
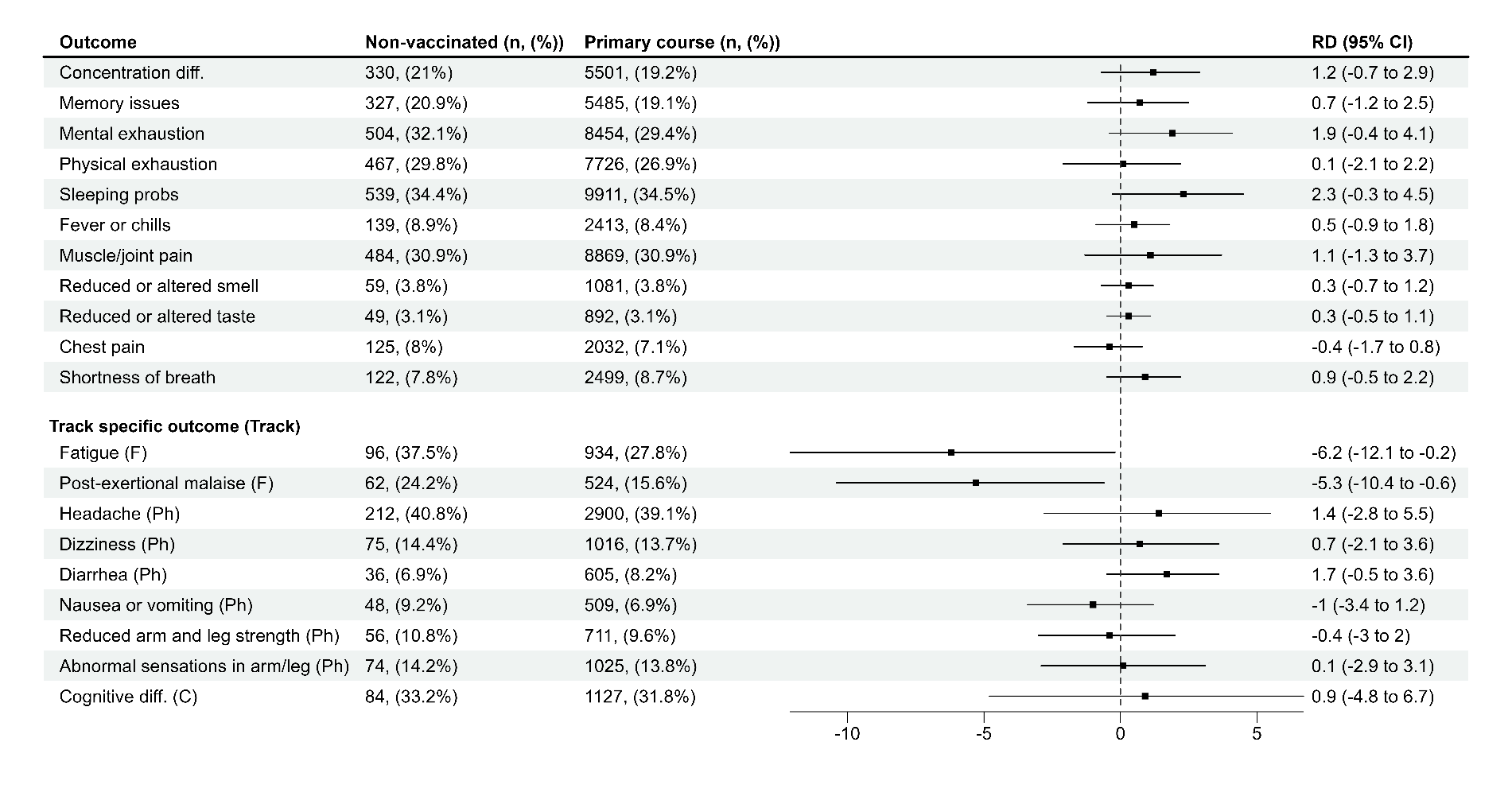
*

# **Supplementary Figure 7** Sensitivity analysis with risk differences (RDs) and 95% confidence intervals (CI) between primary course recipients of BNT162b2 and unvaccinated (ref) participants for self-reported cognitive-, fatigue-related, and physical symptoms.

Total study population N= 30,287 (N_unvaccinated_  = 1,568, N_vaccinated_ = 28,719).

Fatigue track (F): based on the Fatigue Assessment Scale and select questions from Depaul Symptom Questionnaire (N=3,611, n_unvaccinated_ = 256, n_vaccinated_ = 3,355)

Psychiatric track (Ps): based on the Hospital Anxiety and Depression Scale (N=3,803, n_unvaccinated_ = 256, n_vaccinated_ = 3,547)

Physical track (Ph): based on select questions from the 36-Item Short Form Survey Instrument (N=7,934, n_unvaccinated_ = 520, n_vaccinated_ = 7,414)

Cognitive track (C): based on the Cognitive Complaints in Bipolar Disorder Rating Assessment (N=3,793, n_unvaccinated_ = 253, n_vaccinated_ = 3,540)

**
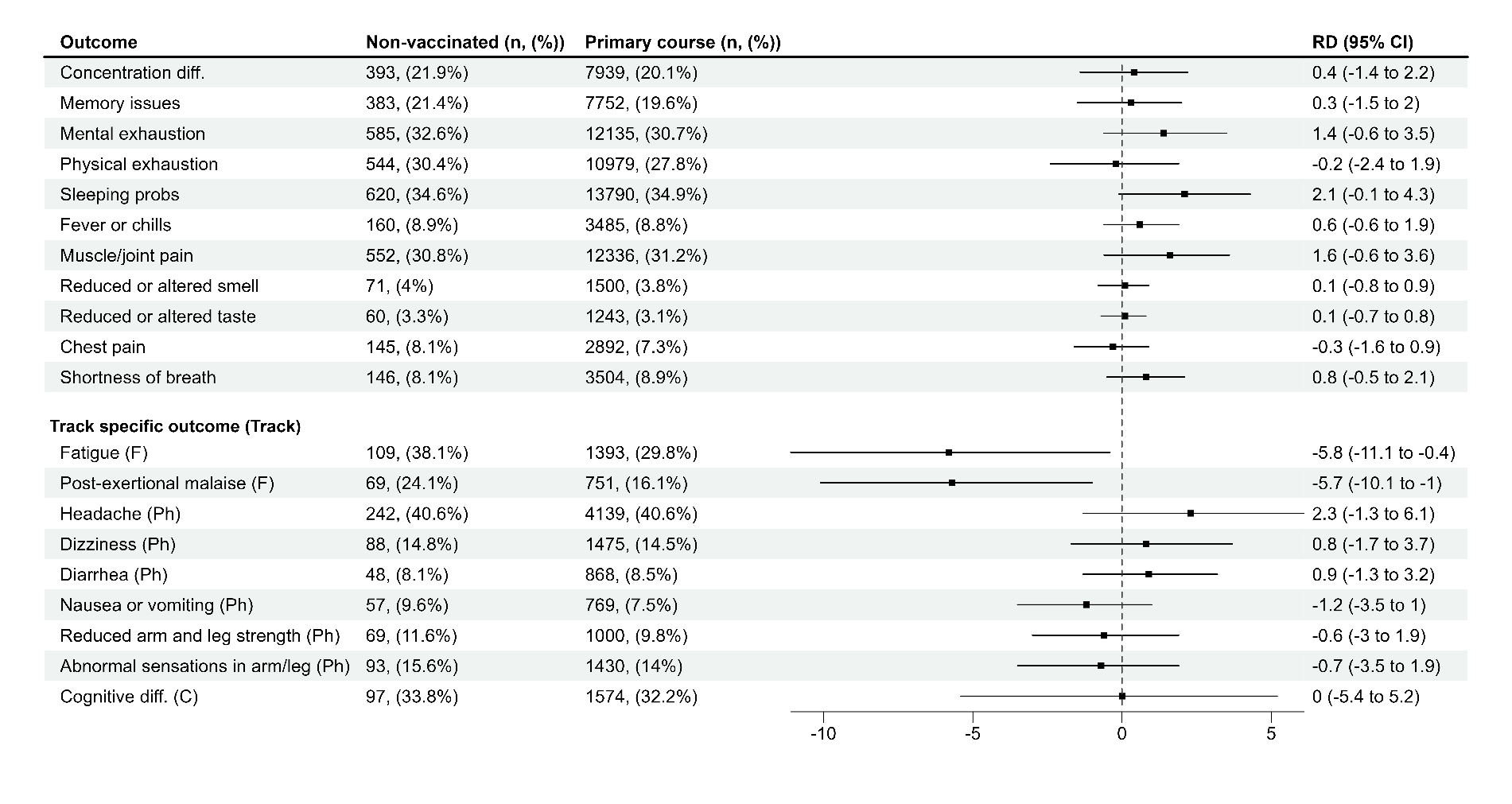
****Supplementary Figure 8** Risk differences (RDs) and 95% confidence intervals (CIs) between 39,562 vaccinated and 1,792 unvaccinated (ref) participants* for self-reported cognitive-, fatigue-related-, and physical symptoms. Vaccinated participants refer to individuals who completed their primary course prior to responding to a follow-up questionnaire. Symptoms were self-reported between September 1, 2021 and May 6, 2022. In variables with missing values, these where treated as a separate category

*This sensitivity analysis includes participants with missing values on smoking habits and alcohol consumption, as well as BMI.

**Supplementary Table 7** Risk differences (RDs) and 95% confidence intervals (CIs) between 16,102 vaccinated and 430 unvaccinated (ref) participants* for self-reported general health symptoms. Vaccinated participants refer to individuals who completed their primary course prior to responding to a follow-up questionnaire. Symptoms were self-reported between September 1, 2021 and November 15, 2021 Adjustments for age, sex, obesity, smoking and alcohol habits, self-reported comorbidities, and Charlson Comorbidity Index score.

| **Outcome** | **Non-vaccinated (n, (%))** | **≤6 weeks** | **7-25 weeks** | **≥ 26 weeks** | **≤ 6 weeks** | **7-25 weeks** | **≥26 weeks** |
| --- | --- | --- | --- | --- | --- | --- | --- |
| Concentration diff. | 80, (18.6%) | 185, (24.5%) | 2552, (18.1%) | 190, (15.4%) | 1.5 (-2.2 to 5.6) | 3.4 (-0.1 to 6.6) | 2.2 (-1.7 to 6.2) |
| Memory issues | 86, (20%) | 133, (17.6%) | 2614, (18.5%) | 231, (18.7%) | -2.4 (-6.6 to 1.8) | 1.1 (-2.4 to 4.6) | 0.1 (-4 to 4.3) |
| Mental exhaustion | 140, (32.6%) | 310, (41%) | 3954, (28%) | 302, (24.4%) | 0.6 (-4.1 to 5.4) | 1.7 (-2.2 to 5.7) | 2.2 (-2.7 to 7.3) |
| Physical exhaustion | 125, (29.1%) | 278, (36.8%) | 3787, (26.8%) | 338, (27.3%) | 5.5 (0.1 to 10.6) | 0.8 (-3.1 to 4.6) | 0.9 (-4 to 5.4) |
| Sleeping probs | 157, (36.5%) | 270, (35.7%) | 4775, (33.8%) | 432, (34.9%) | 0.8 (-5.1 to 6.2) | -0.7 (-5.2 to 3.7) | 0.7 (-4.4 to 5.6) |
| Fever or chills | 33, (7.7%) | 106, (14%) | 1035, (7.3%) | 79, (6.4%) | 4.3 (1.2 to 7.1) | 0.9 (-1.3 to 3.1) | 0.7 (-1.9 to 3.5) |
| Muscle/joint pain | 113, (26.3%) | 238, (31.5%) | 4587, (32.5%) | 427, (34.5%) | 10.2 (4.8 to 15.4) | 6.1 (1.9 to 10.3) | 5.4 (0.5 to 10.3) |
| Reduced or altered smell | 14, (3.3%) | 34, (4.5%) | 520, (3.7%) | 45, (3.6%) | 1 (-0.9 to 3) | 1 (-0.5 to 2.3) | 0.5 (-1.4 to 2.3) |
| Reduced or altered taste | 6, (1.4%) | 23, (3%) | 422, (3%) | 45, (3.6%) | 1.4 (0 to 3) | 1.8 (0.8 to 2.8) | 2.1 (0.7 to 3.4) |
| Chest pain | 26, (6%) | 54, (7.1%) | 872, (6.2%) | 87, (7%) | 0.2 (-2.6 to 2.8) | 0.5 (-1.9 to 2.7) | 1.2 (-1.4 to 3.7) |
| Shortness of breath | 30, (7%) | 49, (6.5%) | 1149, (8.1%) | 145, (11.7%) | 1 (-2.5 to 4.3) | 0.8 (-1.7 to 3.3) | 1.9 (-0.9 to 4.7) |

*****Restricted to participants who responded to the baseline questionnaire before November 15^th^, 2021 (pre-Omicron).

#
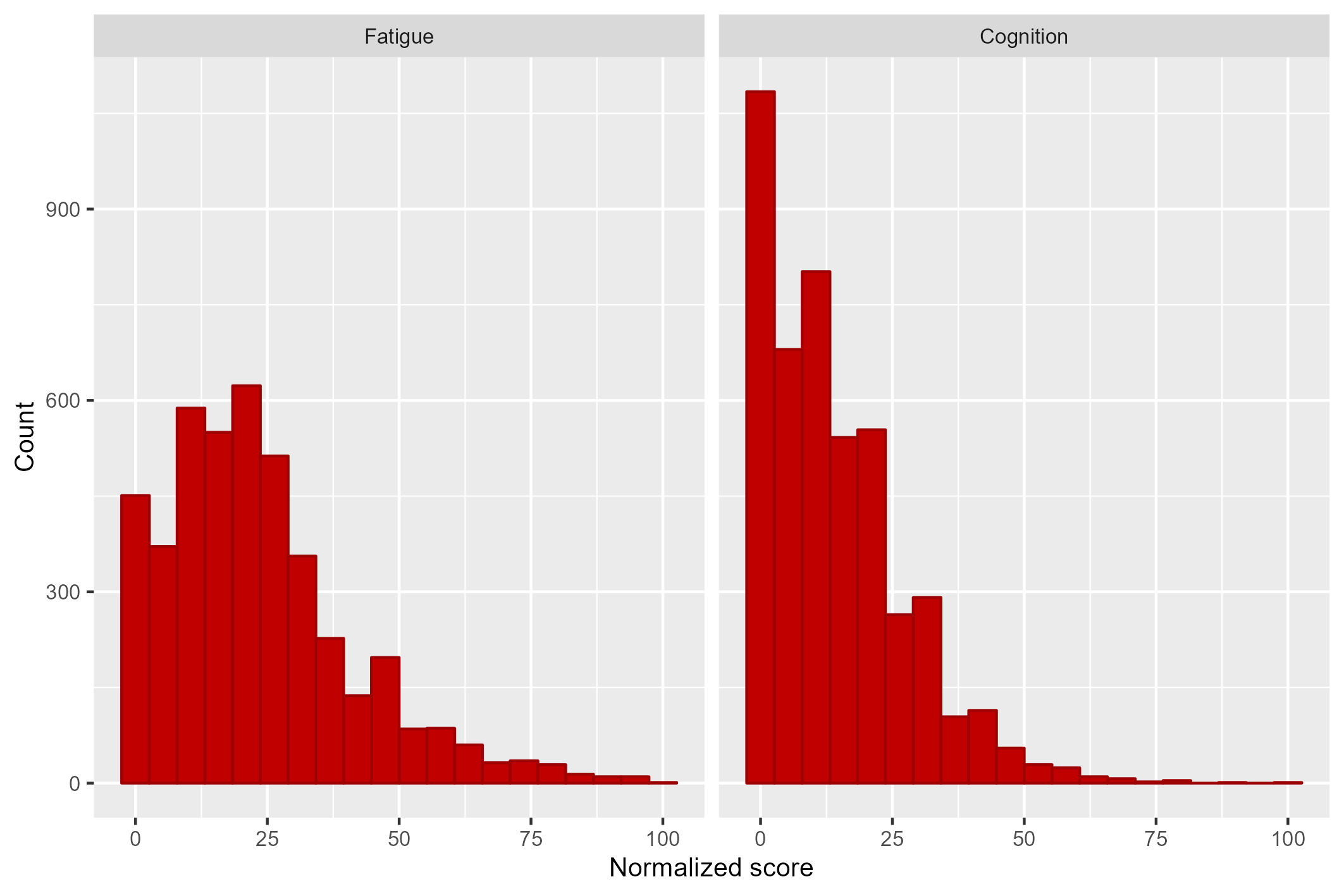
**Supplementary Figure 9:** Distribution of fatigue and cognition scores.

# **Supplementary Note 1** Description of Poisson Regression Models

**Methods**

We further explored the relationship between vaccination status and fatigue, and cognitive scores by fitting two separate adjusted Poisson regression models using the total score from questions about general fatigue, or cognitive difficulties. Poisson regression was used due to the positively skewed distribution of scores and non-negative integers. In these models, we included vaccination status as an explanatory variable together with all of the possible confounders (age, sex, obesity, smoking and alcohol habits, self-reported comorbidities, and Charlson Comorbidity Index score) and computed profile likelihood confidence intervals. As the Depaul Symptom Questionnaire (DSQ) scoring guidelines for post-exertional malaise (PEM) do not allow for the computation of a total score, PEM was not included in this analysis.

**Results**

The fitted Poisson models did not show significant positive associations between vaccination status and fatigue or cognition scores, i.e., we did not observe an increase in scores for vaccinated compared to unvaccinated individuals. The distribution of these normalized scores are available in Figure S5. Primary course recipients had a non-significant 1.0% (95% CI -2.0% to 3.0%) increase in cognition score, compared to unvaccinated individuals. However, we did observe a significant 8.0% (95% CI -10.0% to -5.0%) decrease in fatigue score associated with vaccination.
