# Supplementary material for: A register and questionnaire study of long-term general health symptoms following SARS-CoV-2 vaccination in Denmark
Source: NPJ Vaccines. 2024 Mar 4;9:52. doi: 10.1038/s41541-024-00844-w (PMC10912726; doi:10.1038/s41541-024-00844-w)
Supplement: Supplementary file 2 — Reporting Summary [file 41541_2024_844_MOESM2_ESM.pdf]

nature portfolio

Corresponding author(s): Elisabeth O'Regan
Last updated by author(s): January 9 2024

Reporting Summary

Nature Portfolio wishes to improve the reproducibility of the work that we publish. This form provides structure for consistency and transparency in reporting. For further information on Nature Portfolio policies, see our Editorial Policies and the Editorial Policy Checklist.

Statistics

For all statistical analyses, confirm that the following items are present in the figure legend, table legend, main text, or Methods section.

n/a	Confirmed	
☐	☒	The exact sample size (*n*) for each experimental group/condition, given as a discrete number and unit of measurement
☐	☒	A statement on whether measurements were taken from distinct samples or whether the same sample was measured repeatedly
☐	☒	The statistical test(s) used AND whether they are one- or two-sided<br>*Only common tests should be described solely by name; describe more complex techniques in the Methods section.*
☐	☒	A description of all covariates tested
☐	☒	A description of any assumptions or corrections, such as tests of normality and adjustment for multiple comparisons
☐	☒	A full description of the statistical parameters including central tendency (e.g. means) or other basic estimates (e.g. regression coefficient) AND variation (e.g. standard deviation) or associated estimates of uncertainty (e.g. confidence intervals)
☐	☒	For null hypothesis testing, the test statistic (e.g. *F*, *t*, *r*) with confidence intervals, effect sizes, degrees of freedom and *P* value noted<br>*Give P values as exact values whenever suitable.*
☒	☐	For Bayesian analysis, information on the choice of priors and Markov chain Monte Carlo settings
☒	☐	For hierarchical and complex designs, identification of the appropriate level for tests and full reporting of outcomes
☐	☒	Estimates of effect sizes (e.g. Cohen's *d*, Pearson's *r*), indicating how they were calculated

Our web collection on statistics for biologists contains articles on many of the points above.

Software and code

Policy information about availability of computer code

Data collection	The web-based questionnaires used for data collection were created in SurveyXact (www.surveyxact.dk). The SurveyXact system is used directly online and no version number exists.
Data analysis	All data analysis was conducted in R (version 4.1.3 and 4.2.2). The R packages "riskCommunicator" (v1.0.1) and "forestploter" (v0.2.3) was used.

For manuscripts utilizing custom algorithms or software that are central to the research but not yet described in published literature, software must be made available to editors and reviewers. We strongly encourage code deposition in a community repository (e.g. GitHub). See the Nature Portfolio guidelines for submitting code & software for further information.

Data

Policy information about availability of data

All manuscripts must include a data availability statement. This statement should provide the following information, where applicable:

- Accession codes, unique identifiers, or web links for publicly available datasets
- A description of any restrictions on data availability
- For clinical datasets or third party data, please ensure that the statement adheres to our policy

The data are becoming- or are already available for research upon reasonable request to Statens Serum Institut and within the framework of the Danish data

protection legislation and any required permission from relevant authorities. EO affirms that the manuscript is an honest, accurate, and transparent account of the present study; that no important aspects of the study have been omitted; and that any discrepancies from the study have been planned and explained.

Human research participants

Policy information about studies involving human research participants and Sex and Gender in Research.

Reporting on sex and gender	Sex was adjusted for in the data analysis as a potential confounder and effect modifer of the effect of COVID-19 vaccination on long-term physical, cognitive and fatigue related adverse events. Gender was not used in this study as in could not be asertained from the EFTER-COVID questionnaire. Sex was identified from the unique identifier (CPR-number) in the Danish Civil Registration System assigned to all Danish residents.
Population characteristics	The study cohort consisted of 36,436 participants. Vaccinated participants comprised 95.6% (n = 34,868) of responses, whereas unvaccinated comprised 4.3% (n = 1,568). There was a greater proportion of female participants in both unvaccinated (71.0%) and vaccinated (58.6%) groups, and roughly half of participants had a higher education of ≥2 years among both unvaccinated and vaccinated groups (Table 1). Among the vaccinated, 82.4% (n = 28,719) had received two doses of BNT162b2, 13.4% (n = 4,660) two doses of MRNA-1273, 4.0% (n = 1,394) ChAdOx1 for dose 1 and an mRNA vaccine for dose 2, 0.2% (n = 78) one dose of Ad26.COV2.S, 0.04% (n = 13) two doses of ChAdOx1, and 0.01% (n = 4) mixed mRNA vaccines (Figure S3, Panel B).
Recruitment	Invitations to the EFTER COVID survey were initated by registered RT-PCR test and access to the national digital communication system (92% of population). Test-negative controls were randomly selected among all Danish residents who had recieved a negative test (and no positive) using incidence density sampling on the test date with a ratio of 2:3 between test-positive and -negative persons. This ratio was chosen to compensate for a lower expected response rate among controls compared to cases. Participation bias may have occurred, where individuals living with poor health or adverse events from covid-19 vaccination may have taken more interest in participating along with individuals with strong belief concerning vaccination. Alternatively, some individuals living with poor health may have felt too poorly to participate. This could potentially lead to over- or underestiamtion of the risk of adverse events , respectively. However, given that the survey does not ask about symptoms in relation to COVID-19 vaccines--but rather symptoms experienced within the past 14 days, this study design reduces concern about self-selection and recall bias. Furthermore there is a risk for non-response bias among the unvaccinated. At the time of the study, unvaccinated Danish residents comprised less than 10% aged ≥15 years, and thus symptom experiences might not be fully captured within our sample of individuals who chose to participate in the EFTER COVID survey. However, this reflects the reality of studying COVID-19 vaccine safety in countries where the majority of the adult population has completed the primary course. Further participation bias may be of concern, as never having taken a PCR test for SARS-CoV-2 is associated with being unvaccinated in Denmark, and invitations to the survey hinged on testing.
Ethics oversight	This study was performed as a surveillance study as part of the governmental institution Statens Serum Institut's (SSI) advisory tasks for the Danish Ministry of Health. SSI's purpose is to monitor and fight the spread of disease in accordance with section 222 of the Danish Health Act. According to Danish law, national surveillance activities carried out by SSI do not require approval from an ethics committee.

Note that full information on the approval of the study protocol must also be provided in the manuscript.

Field-specific reporting

Please select the one below that is the best fit for your research. If you are not sure, read the appropriate sections before making your selection.

☒ Life sciences ☐ Behavioural & social sciences ☐ Ecological, evolutionary & environmental sciences

For a reference copy of the document with all sections, see nature.com/documents/nr-reporting-summary-flat.pdf

Life sciences study design

All studies must disclose on these points even when the disclosure is negative.

Sample size	The sample size was not pre-determined. This study uses the individuals with no known history of SARS-CoV-2 and these were invited with a ratio of 3:2 compared to test-postives. Hence, the final sample size is based on the number of individuals with a positive RT-PCR tests in the study period.
Data exclusions	Apart from excluding test-positives for SARS-CoV-2, we excluded individuals who believed they previously had SARS-CoV-2. This included individuals who self-reported that they had previously obtained a seropositive result for SARS-CoV-2 or tested positive on a home rapid antigen test. Furthermore, we did not include individuals who had received incomplete vaccination (e.g., a single dose of an mRNA vaccine) or booster doses prior to responding to the follow-up questionnaire. We were not able to examine incomplete vaccination because the vast majority (92%) completed the primary course within six weeks after the first dose, and few participants reported on symptoms within the period after their first dose. We also could not examine booster doses, as boosting occurred at the same time as widespread omicron infections. In addition, individuals with missing values on confounding variables (e.g., height, weight, smoking-, and drinking habits) were excluded. Further information on our inclusion and exclusion criteria is available in Figure S1.
Replication	The present study is a questionnaire study and has not been replicated. Instead, we have compared our results to results from other sources

Replication	and found them reasonably similar. An English translation of the survey has previously been made available for others to use if they wish to repeat the study.
Randomization	All persons who had recieved a positive PCR results within the study period were invited to participate in the EFTER COVID survey. Controls were randomly selected among persons who had recieved a negative PCR result using incidence density sampling on the test date with a ratio of 2:3 between test-positive and -negative persons. In the present study we only use the test-negative persons.
Blinding	Blinding was not relevant, since this is an observational study, where partcipants were invited based on test status (case or control).

Reporting for specific materials, systems and methods

We require information from authors about some types of materials, experimental systems and methods used in many studies. Here, indicate whether each material, system or method listed is relevant to your study. If you are not sure if a list item applies to your research, read the appropriate section before selecting a response.

Materials & experimental systems

n/a	Involved in the study
☒	☐ Antibodies
☒	☐ Eukaryotic cell lines
☒	☐ Palaeontology and archaeology
☒	☐ Animals and other organisms
☒	☐ Clinical data
☒	☐ Dual use research of concern

Methods

n/a	Involved in the study
☒	☐ ChIP-seq
☒	☐ Flow cytometry
☒	☐ MRI-based neuroimaging
